# Nipagin-Functionalized Porphyrazine and Phthalocyanine—Synthesis, Physicochemical Characterization and Toxicity Study after Deposition on Titanium Dioxide Nanoparticles P25

**DOI:** 10.3390/molecules26092657

**Published:** 2021-05-01

**Authors:** Dariusz T. Mlynarczyk, Daniel Ziental, Emil Kolasinski, Lukasz Sobotta, Tomasz Koczorowski, Jadwiga Mielcarek, Tomasz Goslinski

**Affiliations:** 1 Chair and Department of Chemical Technology of Drugs, Poznan University of Medical Sciences, Grunwaldzka 6, 60-780 Poznań, Poland; emil.kolasinski95@gmail.com (E.K.); tkoczorowski@ump.edu.pl (T.K.); tomasz.goslinski@ump.edu.pl (T.G.); 2 Chair and Department of Inorganic and Analytical Chemistry, Poznan University of Medical Sciences, Grunwaldzka 6, 60-780 Poznań, Poland; dziental@ump.edu.pl (D.Z.); lsobotta@ump.edu.pl (L.S.); jmielcar@ump.edu.pl (J.M.)

**Keywords:** phthalocyanine, porphyrazine, Linstead macrocyclization, Microtox, singlet oxygen, titanium dioxide

## Abstract

Aza-porphyrinoids exhibit distinct spectral properties in UV-Vis, and they are studied in applications such as photosensitizers in medicine and catalysts in technology. The use of appropriate peripheral substituents allows the modulation of their physicochemical properties. Phthalocyanine and sulfanyl porphyrazine octa-substituted with 4-(butoxycarbonyl)phenyloxy moieties were synthesized and characterized using UV-Vis and NMR spectroscopy, as well as mass spectrometry. A comparison of porphyrazine with phthalocyanine aza-porphyrinoids revealed that phthalocyanine macrocycle exhibits higher singlet oxygen generation quantum yields, reaching the value of 0.29 in DMF. After both macrocycles had been deposited on titanium dioxide nanoparticles P25, the cytotoxicities and photocytotoxicities of the prepared materials were studied using a Microtox^®^ acute toxicity test. The highest cytotoxicity occurred after irradiation with a red light for the material composed of phthalocyanine deposited on titania nanoparticles.

## 1. Introduction

Porphyrazines (Pzs) are synthetic aza-analogues of porphyrins, which do not occur naturally [1]. Their unique physicochemical properties result from macrocyclic ring presence, composed of four pyrrole rings linked by azamethine bridges. Pzs that contain four benzo units annulated to the ring are termed phthalocyanines (Pcs). The advantageous properties of Pzs and Pcs include their electrochemical activity, strong light absorption in the UV-Vis range, and the ability to mediate singlet oxygen production, which is beneficial for photodynamic therapy (PDT) applications. All of the abovementioned characteristics allow the application of Pzs and Pcs not only in medicine, but also as sensors and catalysts in chemistry and technology.

Unsubstituted Pz and Pc rings are non-polar and prone to aggregation, which adversely affects some of their properties, such as solubility and singlet oxygen generation. This is the primary reason why many new aza-porphyrinoids with diverse substituents at peripheral positions have been synthesized. It has been noted that, among new Pz derivatives, those that are sulfanyl-based exhibit good solubility, interesting optical, photochemical, and electrochemical properties [2,3,4,5,6], as well as high photocytotoxicities against cancer cells [7]. Further, 4-hydroxyphenyl moiety has been repeatedly described in conjunction with various aza-porphyrinoids as a peripheral substituent or a linker allowing it to obtain more rebuild molecules. An interesting study in this field was presented by Wöhrle et al., who synthesized a metal-free Pc substituted in peripheral positions with 4-carbonylphenyloxy groups [8]. Recently, Taşkın et al. achieved silicon phthalocyanines (SiPcs), substituted axially with different methyl, ethyl, prop-1-yl, and but-1-yl esters of 4-hydroxybenzoic acid [9]. The resulting series of SiPcs was evaluated in terms of photocytotoxicity against *Staphylococcus mutants*, a known dental pathogen. It is worth noting that SiPc substituted with prop-1-ylparaben moiety was found to be the most active against the bacteria’s planktonic form, whereas no significant activity against the biofilm form was observed. In another study, Masilela et al. synthesized an unsymmetrical zinc(II) phthalocyanine (ZnPc) with one nipagin substituent and six 2-(diethylamino)ethylsulfanyl groups in the periphery [10]. This Pz was then used to produce polystyrene nanofibers that were tested for their photodynamic activity against *Staphylococcus aureus* and found to be effective. Lately, Vashurin et al. reported the synthesis of symmetrical A_4_ type cobalt phthalocyanines tetra- or octa-substituted with nipagins in β-positions [11,12]. The macrocycles were then successfully tested as photocatalysts in the oxidation of sulfur-containing organic compounds.

Therefore, the presence of an aromatic macrocyclic ring of a highly hydrophobic nature results in low solubility of such molecules in aqueous systems and, thus, in biological fluids. Many studies have presented the use of specific carriers modifying the properties of porphyrinoids and allowing their application in stable suspensions in water. This method simultaneously increases their application in targeted therapy whilst reducing their intrinsic toxicity [13,14,15]. The delivery agents tested to the greatest extent are liposomes [16,17], metal and metal oxide nanoparticles [18,19,20], and polymeric nanomaterials [21]. Various connections of porphyrinoids with nanoparticles and supramolecules have been studied so far. For example, Baugh et al. synthesized a series of Pcs, including unsymmetrical A_3_B type Pc containing 4-hydroxybenzoic acid moieties in one of their β-positions and *tert*-butylphenyl or functionalized ketals in the other three positions, and studied the action of the system after irradiation [22]. In another study, Rotas et al., synthesized silicon Pc functionalized with *tert*-butyl groups in the periphery and axially substituted with one or two azafullerenes via 4-hydroxybenzoic linkers, and studied the spectral properties of such a system [23]. Mphuthi et al. obtained a material composed among others of metal-free Pc and Fe_3_O_4_ nanoparticles, which enabled and potentiated the sensing of dopamine [24]. In another study, ceria nanoparticles were used as a support for the 5,10,15,20-tetrakis(4-carboxyphenyl) porphyrin [25]. The obtained hybrid material was studied as a catalyst mimicking peroxidase activity, and revealed its activity for glucose detection. Dube et al. combined the designed Pcs with Fe_3_O_4_/SiO_2_ nanoparticles with an amide bond using an aminopropylsilyl linker [26]. The proposed approach gave access to functionalized nanoparticles of potent antimicrobial activity. In another study, photoactivity increase was also observed for polymerized iron phthalocyanine combined with zinc oxide nanoparticles. The obtained material revealed catalytic activity in the photo-Fenton reaction of potential utility for the efficient decomposition of emerging environmental pollutants.

Among other nanoparticles, titanium dioxide (TiO_2_) exhibits special properties. These nanostructures can act as carriers for porphyrinoids or can potentiate the effectiveness of irradiated photosensitizers due to the ability to transfer energy between TiO_2_ and a photoactive molecule [27]. Moreover, TiO_2_ nanoparticles reveal photoactivity only when they are irradiated with UV light and, therefore, are considered relatively non-toxic [28].

In the presented study, Pc and sulfanyl Pz substituted with eight 4-(butoxycarbonyl)phenyloxy moieties were obtained and characterized using UV-Vis and nuclear magnetic resonance (NMR) spectroscopy, mass spectrometry (MS), as well as subjected to photochemical studies in order to determine singlet oxygen quantum yield generation. Both macrocycles were deposited on commercially available P25 titania nanoparticles, and the toxicities of obtained materials were assessed using a Microtox^®^ acute toxicity test.

## 2. Results and Discussion

### 2.1. Synthesis and Characterization

**Scheme 1:**
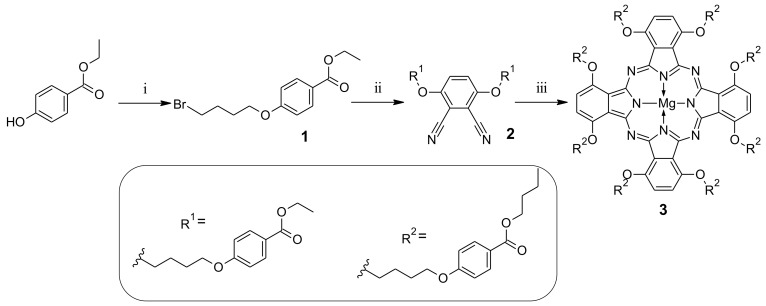
Synthetic route leading to symmetrical phthalocyanine derivative **3**: (**i**) 1,4-dibromobutane, K_2_CO_3_, DMF, rt, 72 h; (**ii**) 3,6-dihydroxybenzene-1,2-dicarbonitrile, K_2_CO_3_, DMF, rt, 20 h; (**iii**) Mg(BuO)_2_, BuOH, 120 °C, 20 h.

The synthetic route leading to new macrocycles appended with 4-hydroxybenzoic moieties started from the alkylation reaction of ethyl 4-hydroxybenzoate with 1,4-dibromobutane (Scheme 1). The obtained ethyl 4-(4-bromobutoxy)benzoate (**1**) was then used in the syntheses of phthalonitrile derivative **2**, that was a precursor of the Pc. Thus, **1** was stirred at room temperature with 3,6-dihydroxybenzene-1,2-dicarbonitrile in *N*,*N*-dimethylformamide (DMF) with K_2_CO_3_ for 20 h to give **2** in a 68% yield after purification. Next, phthalonitrile derivative **2** was used in the Linstead macrocyclization reaction in butan-1-ol using magnesium butan-1-olate as a base towards Pc **3** with a low 2.4% yield by adapting a previously published methodology [29,30]. During cyclotetramerization, the transesterification reaction of ethyl to butyl moieties took place within the ester moieties.

**Scheme 2:**
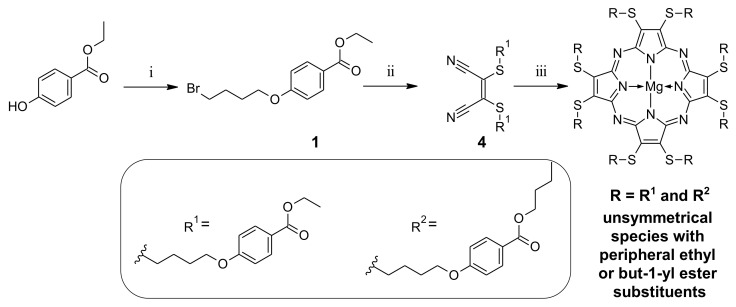
Synthetic route leading to sulfanyl porphyrazine: (**i**) 1,4-dibromobutane, K_2_CO_3_, DMF, rt, 72 h; (**ii**) dimercaptomaleonitrile sodium salt hydrate, K_2_CO_3_, DMF, 50 °C, 20 h; (**iii**) Mg(BuO)_2_, BuOH, 120 °C, 20 h.

Parallelly, compound **1** was subjected to the alkylation reaction with dimercaptomaleonitrile sodium salt and potassium carbonate in DMF at 50 °C for 20 h (Scheme 2), which allowed us to synthesize maleonitrile derivative **4** in a 49.5% yield. Next, compound **4** was used in the macrocyclization reaction towards sulfanyl Pz derivative by adapting a previously published methodology [31]. The macrocyclic product was characterized by NMR and MS techniques. Analysis indicated that the ethyl ester moieties underwent only partial transesterification (see Figures S11–S13 in the Supplementary Materials). It is worth noting that the but-1-yl ester substituted Pz derivative was also not obtained during the macrocyclization reaction when an eightfold molar excess of magnesium butan-1-olate was applied, or when the macrocyclic product with mixed ethyl/but-1-yl periphery was refluxed in butan-1-ol (also in the presence of 1,8-diazabicyclo(5.4.0)undec-7-ene - DBU as a base).

**Scheme 3:**
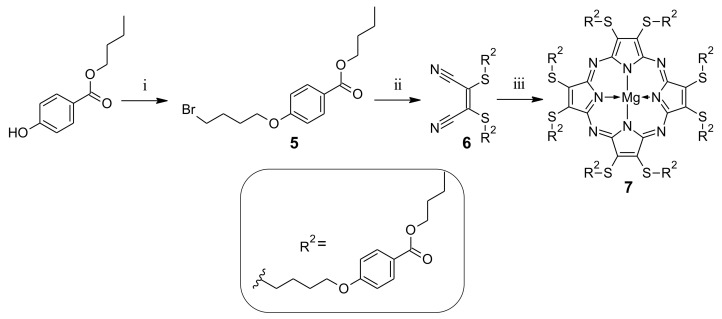
Synthetic route leading to novel sulfanyl porphyrazine **7**: (**i**) 1,4-dibromobutane, K_2_CO_3_, DMF, rt, 72 h; (**ii**) dimercaptomaleonitrile sodium salt hydrate, K_2_CO_3_, DMF, 50 °C, 20 h; (**iii**) Mg(BuO)_2_, BuOH, 120 °C, 20 h.

As the butyl and ethyl groups could not be unambiguously assigned, and the HPLC results gave clear information that there is only one compound present in the sample, a different synthetic approach was proposed. But-1-yl 4-hydroxybenzoate was proposed as a starting material in place of ethyl 4-hydroxybenzoate **1** to avoid the problem of transesterification reaction (Scheme 3).

**Scheme 4:**
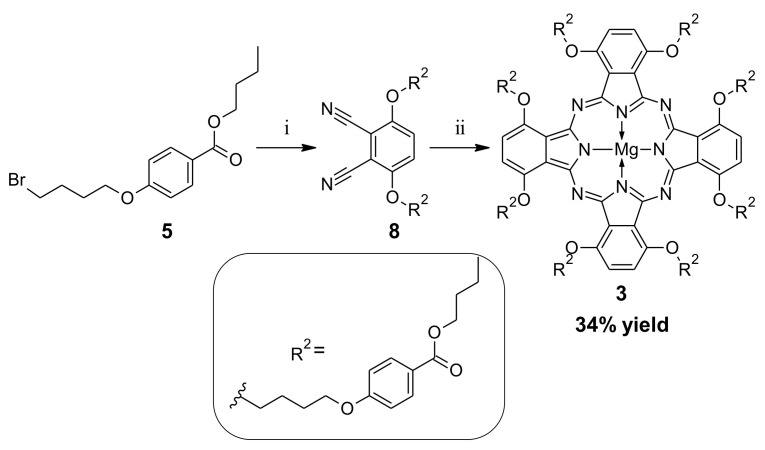
Alternative synthetic route leading to **3**: (**i**) 3,6-dihydroxybenzene-1,2-dicarbonitrile, K_2_CO_3_, DMF, rt, 20 h; (**ii**) Mg(BuO)_2_, BuOH, 120 °C, 20 h.

Following the reaction conditions elaborated for **1**, but-1-yl 4-hydroxybenzoate was used in the alkylation reaction, with an excess of 1,4-dibromobutane towards compound **5**. This product was isolated after column chromatography with an average 25% yield. It was because of the formation of the dimeric side product **5D** with a 43% yield (see Figures S16–S17). The formation of dimers in similar conditions has also been described by other authors in the literature [32]. Next, **5** was purified using column chromatography and subjected to the alkylation reaction with dimercaptomaleonitrile sodium salt hydrate towards dimercaptomaleonitrile derivative **6**. Dimercaptomaleonitrile derivative **6** was applied in the macrocyclization reaction with magnesium butan-1-olate in refluxing butan-1-ol, leading to sulfanyl Pz **7** in a moderate 32% yield. The course of macrocyclization reaction, with the use of but-1-yl ester of mercaptomaleonitrile derivative, prompted us to reconsider the alternative synthetic approach for the previously obtained Pc **3** (Scheme 4). Thus, derivative **5** was subjected to the alkylation reaction with 3,6-dihydroxybenzene-1,2-dicarbonitrile towards phthalonitrile derivative **8**, which was isolated with a 76% yield. Phthalonitrile derivative **8** was then used in the Linstead macrocyclization reaction, with magnesium butan-1-olate as a base in butan-1-ol to give the desired Pc macrocycle **3**. The macrocyclization reaction with **8** was much more efficient than with the previously applied phthalonitrile derivative **2**, and led to Pc **3** in a moderate 34% yield.

Pc **3** and Pz **7**, were soluble in organic solvents, such as dichloromethane (DCM), toluene, tetrahydrofuran, acetonitrile, acetone, DMF, dimethyl sulfoxide (DMSO), and pyridine. Neither of them were soluble in methanol (MeOH) and other polar solvents. Differences were observed when attempting to precipitate the macrocycles from DCM by the slow addition of MeOH. At the time, **3** formed a green precipitate, as expected, whereas **7** formed a stable non-transparent blue suspension and could not be purified in this way.

We also attempted to synthesize A_3_B type macrocycles by using either phthalonitrile or maleonitrile derivatives **2**, **4**, **6**, **8**, and an excessive amount of 1,2-dicyanobenzene (ten-fold mol/mol) each time. In each case, only the formation of a tiny amount of A_3_B products was observed in the matrix-assisted laser desorption/ionization (MALDI) mass spectra, but the estimated yields were about ten times lower than for symmetrical analogues. Moreover, A_3_B products could not be isolated in their pure form and the NMR spectra were of low quality (for more information, please refer to Figures S30–S31).

All compounds were subjected to detailed NMR studies (see Figures S3–S23). In the ^1^H NMR spectrum of **1**, signals at 1.29 and 4.25 ppm were assigned to ethyl ester CH_3_ and CH_2_ protons, respectively. The other aliphatic part of the chain, namely 4-bromobutoxy moiety, appeared as four signals at 3.59, 1.96, 1.84 and 4.07 ppm for CH_2_ groups, consecutively, from BrCH_2_ to CH_2_O, which correlate well in the ^1^H-^1^H correlation spectroscopy (COSY) NMR spectrum. The characteristic signals from the *p*-substituted benzene ring protons were found at 7.03 and 7.90 ppm. Derivative **2** shares a similar structural motif with signals at 4.25 and 1.30 ppm assigned to the ethyl chain, and signals at 4.14, 1.91 and 4.23 ppm prescribed to the linker group. The *para*-substituted benzene aromatic protons were found at 7.02 and 7.89 ppm. The dicyanobenzene aromatic protons appeared as a singlet at 7.61 ppm. The carbon atom signals appearing in the ^13^C NMR spectrum were assigned with the use of ^1^H-^13^C heteronuclear single quantum coherence (HSQC) and ^1^H-^13^C heteronuclear multiple-bond correlation spectroscopy (HMBC) spectra for cyano group at 113.5 ppm, dicyanobenzene at 102.8, 120.5, 154.8 ppm, *para*-substituted benzene at 114.4, 122.0, 131.1, 162.4 ppm, and carboxylic group at 165.4 ppm. In the ^1^H and ^1^H-^1^H COSY NMR spectra recorded for Pc **3**, the transesterification reaction within ester moieties was clearly confirmed. Butyl ester protons appeared at 0.85, 1.34, 1.60, and 4.29 ppm, while butylene linker part protons were found at 4.26, 2.31, 2.52, and 5.24 ppm. Eight aromatic protons of the Pc ring appeared as a singlet peak at 7.90 ppm. The characteristic aromatic protons from nipagin moieties were found at 8.09 and 7.04 ppm. The ^1^H NMR spectrum of **4** revealed two groups of signals, resulting from the ethyl group at 1.29 and 4.26 ppm, and from butylene moiety at 4.05, 1.82, and 3.23 ppm. The C2 and C3 linker CH_2_ groups protons appeared as a multiplet in the ^1^H NMR spectrum at 1.82 ppm due to overlapped signals—this multiplet signal correlated in the ^1^H-^13^C HSQC spectrum with two carbon nuclei signals at 26.1 and 27.1 ppm. Benzene ring protons were assigned to signals at 6.99 and 7.89 ppm.

In the ^1^H NMR spectrum of **5**, many similarities to its ethyl analogue **1** were found. The group of signals at 0.86, 1.35, 1.64, and 4.33 ppm was assigned to the butyl protons, while the signals from the butylene linker were found at 4.17, 2.04, 2.41 and 5.51 ppm. The aromatic signals at 7.16 and 8.18 ppm confirming the *para*-substitution come from the protons within the benzene ring. Similarly, in the ^1^H NMR spectrum of **6**, two groups of proton signals can be distinguished, correlating separately in the ^1^H-^1^H COSY spectrum. It concerns signals at 0.92, 1.40, 1.67 and 4.22 ppm from the butyl ester aliphatic hydrogen atoms and 4.07, 1.83, and 3.25 ppm from the butylene linker CH_2_ groups. The characteristic signals coming from the *para*-substituted benzene ring were found at 7.00 and 7.88 ppm. In the ^1^H NMR spectrum of **7**, two groups of signals were distinguished at 0.92, 1.39, 1.66, and 4.21 ppm, confirming the presence of butyl ester protons, and at 4.06, 1.83, and 3.24 ppm, coming from the butylene linker. Signals of protons from the *para*-substituted benzene ring were found at 6.99 and 7.87 ppm. The ^13^C NMR spectrum revealed macrocyclic ring signals at 112.4 and 121.2 ppm. In the ^1^H NMR spectrum of **8**, a group of aliphatic protons correlating in the ^1^H–^1^H COSY at 0.90, 1.38, 1.65, and 4.20 ppm was detected, and which was assigned to butyl ester moiety. Another group of signals, appearing at 4.12, 1.89 and 4.20 ppm, was assigned to the butylene linker. Three signals in the aromatic part of macrocycle **8** were prescribed to the nipagin ring protons (signals at 7.00 and 7.85 ppm) and the 1,2-dicyanobenzene ring (signal at 7.59 ppm).

The ^1^H and ^13^C NMR spectra, as well as the correlations found in the ^1^H-^1^H COSY, ^1^H-^13^C HSQC, and ^1^H-^13^C HMBC spectra for all compounds discussed in this paper, are presented in the Supplementary Materials.

### 2.2. TiO_2_ Deposition and Characterization

Titanium dioxide, mostly a mixture of crystal phases of anatase and rutile, known as P25, has been utilized in many studies as a carrier for photosensitizers [33,34]. It forms stable dispersions for compounds that are insoluble in water and, thus, can be tested and potentially applied. The surface of titanium dioxide and other oxides may be functionalized with porphyrinoids following various methods. Molecules substituted with anchoring groups interact with the surface of the nanoparticles, which results in stable bond formation and allows for energy transfer between both components [35]. Various macrocycles can also be embedded on the formed surface of the oxide nanoparticles during their synthesis [36]. Alternatively, the nanoparticles can be appended with various linkers (i.e., by silylation), which allows for covalent bond formation between the nanoparticles and the photosensitizers [26]. Finally, the interactions could consist in weak bonds between the photosensitizers and the unmodified surface of the nanoparticles, or the chemical groups present on their surfaces after functionalization, i.e., hydrophobic interactions with the long PEG chains [37]. In the presented study, titania nanoparticles with a plethora of hydroxyl surface groups were applied to influence their adsorption properties and enable the formation of hydrogen bonds [38]. The materials were prepared by depositing either **3** or **7** on the surface of TiO_2_ nanoparticles. The solutions of macrocycles were added to titania suspension, sonicated, and mixed until the solvent evaporated, yielding **3**@P25 and **7**@P25, respectively. The resulting materials contain 10% (w/w) of the macrocycles. The particle sizes of the obtained materials were assessed using nanoparticle tracking analysis and compared to the values obtained for unmodified TiO_2_ nanoparticles. The results are summarized in Table 1.

Based on the particle size values measured, strong agglomeration of the nanoparticles was observed. The mean hydrodynamic particle sizes of **3**@P25 (148.4 ± 99.3 nm) and **7**@P25 (159.1 ± 101.2 nm) were similar to unmodified P25 (132.1 ± 85.1 nm), which suggests that the deposition of the macrocycles had only a negligible effect on the titania nanoparticles. Additionally, the calculated polydispersity indices values exceed 0.2, deeming them polydisperse materials [39]. These phenomena may be attributed to the fact that no stabilization was applied to the materials, including applying of surfactants or moieties capable of electrostatic interactions. Nevertheless, such stabilizers could induce some toxic effects of nanoparticles, which are undesirable when the materials could be considered for photodynamic therapy.

### 2.3. Spectral and Photochemical Studies

#### 2.3.1. Absorption and Fluorescence

**Figure 1:**
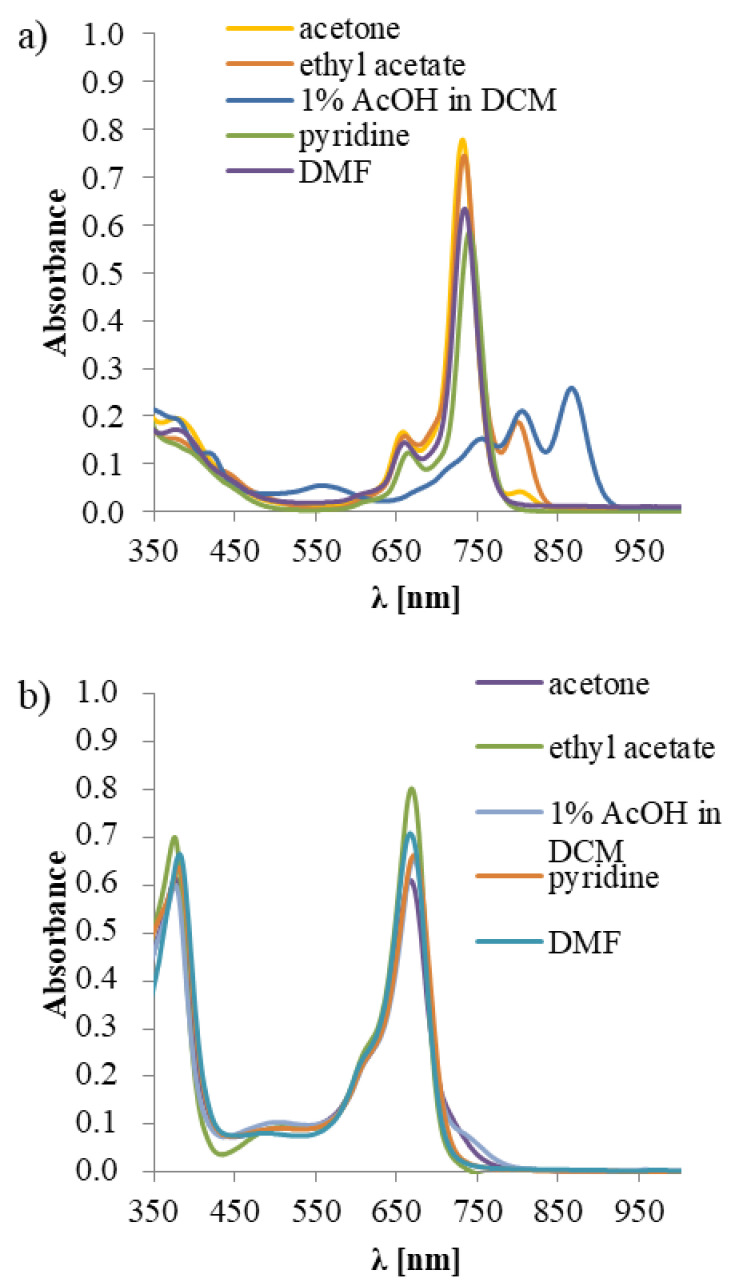
Solvatochromic effects observed in the UV-Vis spectra in various solvents for (**a**) **3**; (**b**) **7**; AcOH—acetic acid, DCM—dichloromethane, DMF—*N*,*N*-dimethylformamide.

Each of the studied macrocyclic compounds possesses two characteristic absorption bands—the B band in the blue region and the Q band in the red region of the UV-Vis spectrum. For macrocycle **3**, a typical Pc absorption spectrum profile (Figure 1a) was observed—B band with a low molar absorption coefficient (ε) and an intensive sharp Q band. An assessment of the effects of different solvents on the UV-Vis absorption properties of **3** was performed. In the acidic solvents mixture composed of 1% acetic acid (AcOH) in dichloromethane, the splitting of the Q band was noted (this process was reversible after adding a base), whereas, in basic pyridine, the Q band remained unsplit (Figure 1a). The observed effects can be explained by the protonation of phthalocyanine meso nitrogen atoms [40].

In the UV-Vis absorption spectrum of Pz **7**, typical and intense B and Q bands were noted. Interestingly, in all studied solvents containing **7**, the Q band remained unsplit (Figure 1b). This observation is in agreement with the data collected by Khelevina and Malyasova, who reported that Pzs reveal low basicity and, therefore, a high concentration of acid has to be used to induce their protonation [41].

**Figure 2:**
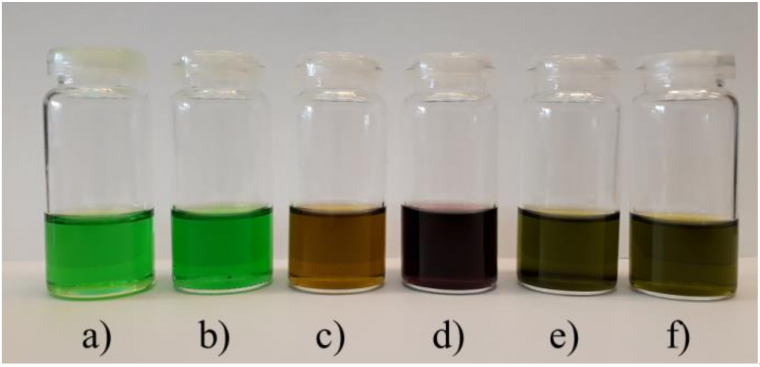
Effect of solvents used on the color of the solution of **3**: (**a**) pyridine, (**b**) *N*,*N*-dimethylformamide, (**c**) dichloromethane, (**d**) dichloromethane + 1% of acetic acid, (**e**) acetone, (**f**) ethyl acetate.

In the emission study of macrocycles **3** and **7**, only Pc **3** presented weak emission properties, with the quantum yields (Φ_FL_) equal to 0.007 and 0.002 in DMF and DMSO, respectively. The drop in Φ_FL_ values noted for **7**, in comparison to the unsubstituted magnesium(II) phthalocyanine (33-fold in DMF and 91-fold in DMSO [42,43]), could be explained by a more developed aromatic periphery in case of Pcs. It has been reported by Kobayashi and co-workers that substitution of Pc at *α* positions (non-peripheral) impacts a fluorescence yield [44]. In the case of Pc **3**, the solvatochromic-like effects [45,46] were observed, which was not the case for Pz **7**. Monomeric form of **3** with a sharp and an intense Q band represents a green color (see Figure 1 and Figure 2). Interestingly, in the UV-Vis profiles of **3** recorded in the dichloromethane, acetone and ethyl acetate a split, and a lowering of the Q band intensity was observed. In these solvents, the Pc presented colors from brown in dichloromethane to rotten green in acetone or ethyl acetate. It proves that the extended aromatic ring of Pc, in comparison to the Pz one, displays a strong solvatochromism, which is further reflected by changes in colors in the applied solvents. It can be also caused by a well-known, and reported in the literature, π-π stacking between molecules, including magnesium(II) phthalocyanines [40]. In the acidic conditions (dichloromethane with 1% of acetic acid), the protonation of macrocycle **3** was observed in the UV-Vis, which manifested by the splitting of the Q band to three counterparts (see Figure 1) and the dark-red color of the solution.

#### 2.3.2. Singlet Oxygen Formation

**Figure 3:**
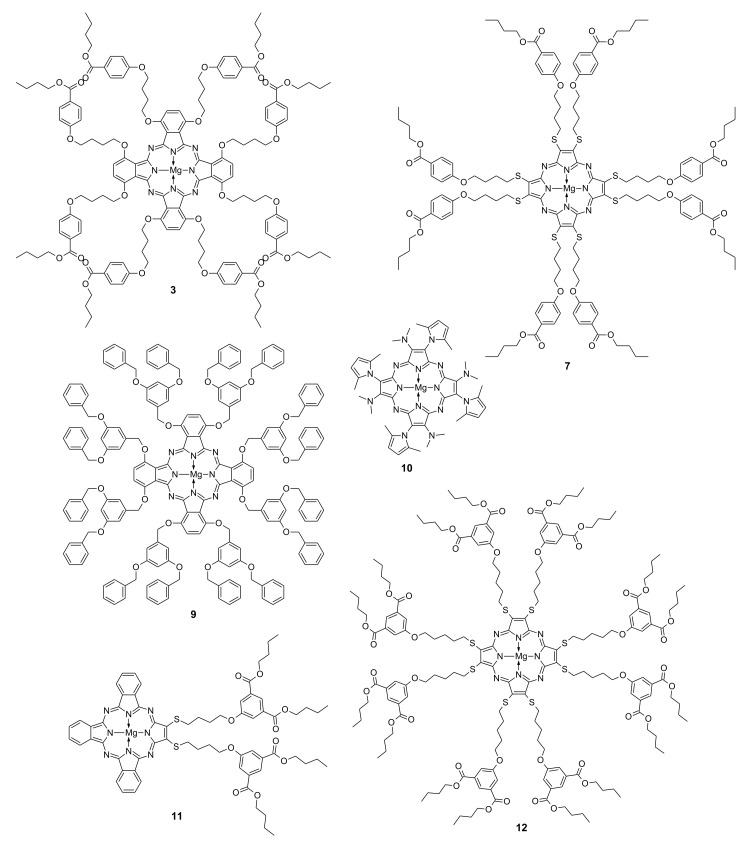
Chemical structures of macrocycles **3**, **7** and **9–12**.

Singlet oxygen (^1^O_2_) is a crucial factor appearing in a type II photodynamic reaction as the result of interaction between light, molecular oxygen, and photosensitizer, and which can be considered as an energy conversion platform. The active oxygen form belongs to reactive oxygen species and is responsible for the effects observed in PDT of cancer, as well as against bacteria and other pathogens. Both studied macrocyclic compounds were able to mediate singlet oxygen generation, which was exemplified by singlet oxygen quantum yield values (Φ_∆_) obtained during measurements performed in various solvents. The herein reported Pc **3** was a more efficient ^1^O_2_ generator, with the Φ_∆_ equal to 0.29 and 0.13 in DMF and DMSO, respectively, in comparison to Pz **7**, which revealed low Φ_∆_ values equal to 0.02 and 0.09 in DMF and DMSO, respectively. These Φ_∆_ values were in agreement with previous studies, in which phthalocyanines were found to be more efficient singlet oxygen generators compared to porphyrazines [47,48], especially sulfanyl porphyrazines [7]. Pc **3** revealed a lower singlet oxygen formation quantum yield in comparison to the reference compound—unsubstituted ZnPc. This could be related to the presence of developed substituents in its non-peripheral positions, which might hamper access of molecular oxygen to the macrocyclic core, whose direct interaction is necessary for ^1^O_2_ formation [49]. A similar phenomenon was noted for Pc **9** (Figure 3) [50]. Due to the presence of large substituents, Pz **7** revealed a decrease in the quantum yield, which was linked to steric hindrance and limited oxygen access to the macrocyclic core. The lowering of the Φ_∆_ value can be clearly seen by comparison of **7** (Φ_∆_ = 0.02, Table 2) and previously reported Pz **10** (Φ_∆_ = 0.14) [48]. The influence of sulfur atoms constituting a bridge between the macrocyclic ring and peripheral substituents should also be mentioned. According to the literature data, the presence of a sulfur atom directly linked to the Pz core results in a strong decrease of singlet oxygen formation yield [51,52]. Lately, we have reported sulfanyl tribenzoporphyrazines bearing isophthaloxy moiety **11**, which was found to generate singlet oxygen with a much lower yield at 0.05 in DMF [42], in comparison to Pc **3**, with Φ_∆_ equal to 0.29, studied here. Measurements performed in DMSO revealed a slighter difference in the values of singlet oxygen generation at 0.13 vs. 0.08 [42] for **3** and **11**, respectively. This observation could be explained by possible quenching of the excited states by π-π interactions between molecules of compound **11**. Lately, we reported studies concerning Pz **12**, similar in structure to molecule **7** studied here. Singlet oxygen quantum yield values for **12** (0.03 in DMF [51,53]) were reported, and are similar to the values obtained for **7**. They indicate that the modification of the linker chain length and the substituents in the periphery of benzene ring had no effect on Φ_∆_, and that it was determined mostly by the central metal ion and the presence of sulfanyl moieties attached to the Pz ring.

#### 2.3.3. Photostability

Singlet oxygen formed during irradiation can also self-destructively contribute to the stability of photosensitizer. Two mechanisms of the macrocyclic system decomposition were recognized: (i) photobleaching, which leads to the formation of low-weight, colorless products, and (ii) phototransformation, which can lead to the modification of the macrocycle [54,55]. Pz **7**, as well as Pc **3**, belong to stable photosensitizers and, after irradiation, decomposed following the photobleaching mechanism. Stable photosensitizers reveal photodecomposition quantum yield (Φ_P_) values at ca. 10^−6^, as categorized by Dilber and co-workers [56]. In this presented study, well-developed peripheries around the macrocyclic cores caused steric hindrances and influenced measured photostabilities in comparison to the reference compound - ZnPc (Table 2). Interestingly, Pc **3**, when dissolved in DMF, revealed Φ_P_ values one order of magnitude higher, which might be linked with the predisposition of magnesium(II) macrocycles to the demetallation reaction [57]. On the other hand, the substituents attached to the phthalocyanine ring in **3** stabilize it in comparison to the unsubstituted magnesium(II) phthalocyanine (in DMF Φ_P_ equal 52.5 × 10^−6^ vs. 327 × 10^−6^ [43], respectively). The electron-donating groups—sulfanyl bridges—were reported to increase photostability [55,58]. The enhancement of photostability could be influenced by the formation of an intracoordination bond between the central magnesium(II) ion and the oxygen atom of the peripheral substituent. We have reported such a phenomenon for alkoxy octa-substituted magnesium(II) phthalocyanine [40].

**Table 2:** Fluorescence (Φ_FL_), photodecomposition (Φ_P_), and singlet oxygen generation (Φ_Δ_) quantum yields of macrocycles **3** and **7**.

Compound	Solvent	Φ_FL_	Φ_P_	Φ_Δ_
**3**	DMF	0.007	52.50 × 10^−6^	0.29
	DMSO	0.002	3.80 × 10^−6^	0.13
**7**	DMF	-	3.08 × 10^−6^	0.02
	DMSO	-	2.66 × 10^−6^	0.09
**ZnPc** (**reference**)	DMF	0.20 [59]	10.2 × 10^−6^ [60]	0.56 [61]
	DMSO	0.17 [59]	3.5 × 10^−6^ [60]	0.67 [61]
**9**	DMF DMSO	0.17 [50] 0.04 [50]	37.5 × 10^−6^ [50] 3.07 × 10^−6^ [50]	0.13 [50] 0.11 [50]
**10**	DMF DMSO	0.046 [48] 0.012 [48]	1.81 × 10^−4^ [48] 1.37 × 10^−4^ [48]	0.14 [48] 0.14 [48]
**11**	DMF DMSO	0.008 [42] 0.002 [42]	48.4 × 10^−6^ [42] 4.84 × 10^−6^ [42]	0.05 [62] 0.08 [42]
**12**	DMF DMSO	- -	6.14 × 10^−6^ [53] -	0.03 [51] -

ZnPc—zinc(II) phthalocyanine, DMF—*N*,*N*-dimethylformamide, DMSO—dimethyl sulfoxide.

### 2.4. Acute Toxicity Assessment

**Figure 4:**
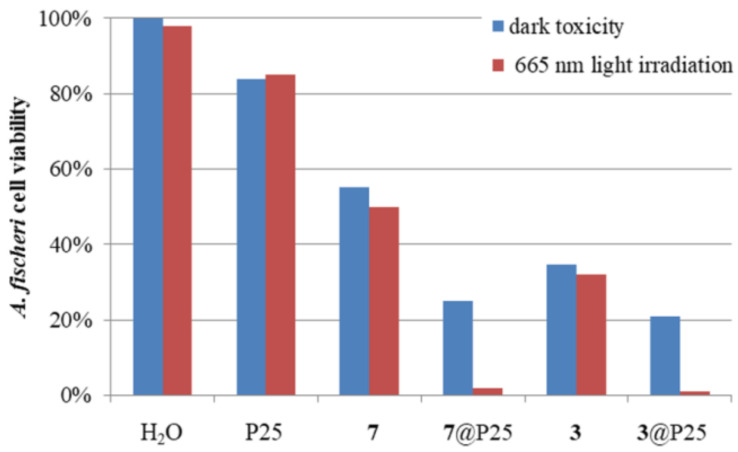
Changes in *A. fischeri* cell viability after 15 min of the experiment when incubated with different materials (P25, **3**@P25, **7**@P25) or compounds (**3**, **7**). The addition of water served as the control experiment (H_2_O).

The obtained macrocycles and titania materials were tested on *Aliivibrio fischeri* to evaluate their acute toxicity and photocytotoxicity. *A. fischeri* is a bacterium that emits bioluminescence which is directly correlated to its metabolism and, thus, its viability [63]. The obtained titania materials, as well as **3** and **7**, were studied in water/DMSO mixtures (1% of DMSO addition). The samples were tested in the dark and upon irradiation with 665 nm light to assess the difference between irradiated and non-irradiated photosensitizers. Additionally, in order to assess the effect of light and P25 nanoparticles, an experiment with studied bacterium irradiated with light in the absence of the photosensitizer was performed, and a study in which unmodified suspension of P25 was added to the sample containing the bacterium. The results are presented in Figure 4.

Initially, the effect of red light on cell viability was measured. It was found that the irradiation had a negligible effect on *A. fischeri*, as cell viability decreased only by 2% (H_2_O in Figure 4). In the case of unmodified P25, the effect was stronger, but still below 20%, which is regarded as non-toxic in a Microtox^®^ test [64]. The compounds **3** and **7** also exhibited significant dark toxicity, which changed only slightly after irradiation. In the case of both materials tested, **3**@P25 and **7**@P25, the dark toxicity reached almost 80%, but the effect was the strongest together with irradiation, reaching nearly a 100% decrease in cell viability. What is more, in the case of the macrocycles deposited on titania, the effect of irradiation was stronger than in the case of macrocycles alone. For Pc derivative **3**, cell viability decreased by 3% upon irradiation, as compared to its dark toxicity, whilst, for **3**@P25, a drop of 20% was observed.

It seems that the high values measured for the dark toxicity of both nanomaterials and, separately, porphyrinoids, are related to the presence of nipagin moieties present in the periphery of the macrocycles, which are known to exhibit strong antimicrobial properties [65].

## 3. Materials and Methods

### 3.1. Materials and Instruments

All reactions were conducted in oven-dried glassware under an argon atmosphere using *Radleys Heat-On*^TM^ heating system. All solvents and reagents were obtained from commercial suppliers and used without further purification, except for dichloromethane, which was distilled before use. All solvents were removed by rotary evaporation at or below 50 °C. Dry flash column chromatography was carried out on Merck silica gel 60, particle size 40–63 µm; reverse phase Fluka C18 silica gel 90; aluminium oxide 90 active neutral (activity stage I) for column chromatography 0.063–0.200 mm, EMD Millipore. Thin-layer chromatography (TLC) was performed on silica gel Merck Kieselgel 60 F_254_ plates and Merck Kieselgel RP-18 60 F_254_s visualized with UV (*λ*_max_ 254 or 365 nm). All mobile phases and solvent mixtures are given in volume-to-volume (v/v) ratio, unless otherwise stated. UV-Vis spectra were recorded on Hitachi UV/VIS U-1900 and Shimadzu PC-160 spectrophotometers. ^1^H and ^13^C NMR spectra were acquired on an AVANCE II Bruker 400 MHz (9.39 T), AVANCE III Bruker 500 MHz (11.74 T), and AVANCE III Bruker 700 MHz (16.44 T) spectrometer at 298 K. Chemical shifts (δ) were reported in parts per million (ppm) and referenced to the residual solvent peak (DMSO-*d_6_*: δ_H_ = 2.50 ppm, δ_C_ = 39.50 ppm; pyridine-*d_5_*: δ_H_ = 8.74, 7.58, 7.22 ppm, δ_C_ = 150.35, 135.91, 123.87 ppm). Coupling constants (*J*) are quoted in Hertz (Hz) and round up to 0.5. The abbreviations s, bs, d, t, q, m refer to singlet, broad singlet, doublet, triplet, quadruplet, and multiplet, respectively. ^1^H and ^13^C resonances were unambiguously assigned based on ^1^H-^1^H COSY, ^1^H-^13^C HSQC and ^1^H-^13^C HMBC experiments. Mass spectra (MS ES, HRMS ES, MALDI TOF) were carried out by the Wielkopolska Center for Advanced Technologies at Adam Mickiewicz University in Poznan and the Department of Inorganic and Analytical Chemistry at Poznan University of Medical Sciences.

### 3.2. Synthesis

#### 3.2.1. Ethyl 4-(4-bromobutoxy)benzoate (**1**)

Ethyl 4-hydroxybenzoate (4.00 g, 24.07 mmol), 1,4-dibromobutane (7.10 mL, 60.18 mmol), and potassium carbonate (33.27 g, 0.24 mol) in 100 mL of anhydrous DMF were stirred at room temperature for 72 h. Next, the reaction mixture was filtered through Celite and washed with DCM. The filtrate was evaporated and the residue was chromatographed using silica gel and DCM:hexane 1:1 as eluent to give 4.340 g of colorless liquid, which was identified as **1** (yield 72%). R*_f_* (DCM:hexane 1:1) 0.38. UV-Vis (DCM): *λ_max_*, nm (log *ε*) 257 (4.38). ^1^H NMR (400 MHz, DMSO-*d*_6_) δ 7.90 (d, *J* = 9.0 Hz, 2H), 7.03 (d, *J* = 9.0 Hz, 2H), 4.25 (q, *J* = 7.0 Hz, 2H), 4.07 (t, *J* = 6.5 Hz, 2H), 3.59 (t, *J* = 6.5 Hz, 2H), 1.96 (m, 5H), 1.84 (m, 2H), 1.29 (t, *J* = 7.0 Hz, 3H). ^13^C NMR (101 MHz, DMSO-*d*_6_) δ 165.3, 162.4, 131.1, 122.1, 114.3, 67.0, 60.3, 34.6, 29.0, 27.2, 14.2. HRMS (ESI) *m/z* found: 301.0435, [M+H]^+^ C_13_H_18_BrO_3_ requires 301.0439.

#### 3.2.2. 1,2-Dicyano-3,6-bis [4-(4-ethoxycarbonylphenoxy)butyloxy]benzene (**2**)

Compound **1** (4.34 g, 14.41 mmol), 3,6-dihydroxybenzene-1,2-dicarbonitrile (1.05 g, 6.55 mmol), and potassium carbonate (4.52 g, 32.75 mmol) were stirred in anhydrous DMF (45 mL) at room temperature for 20 h. Next, the reaction mixture was poured on ice, the precipitate was filtered, washed with methanol, and recrystallized from ethanol to give 118 mg of **2** as white powder (yield 68%). R*_f_* (DCM) 0.11. UV-Vis (DCM): *λ_max_*, nm (log *ε*) 227 (4.47), 252 (4.59), 348 (3.78).^1^H NMR (500 MHz, DMSO-*d*_6_) δ 7.89 (d, *J* = 8.5, 4H), 7.61 (s, 2H), 7.02 (d, *J* = 8.5, 4H), 4.25 (m, 4H), 4.23 (m, 4H), 4.14 (m, 4H), 1.91 (m, 8H), 1.30 (t, *J* = 7.0, 6H). ^13^C NMR (126 MHz, DMSO-*d*_6_) δ 165.4, 162.4, 154.8, 131.1, 122.0, 120.5, 114.4, 113.5, 102.8, 69.5, 67.4, 60.3, 25.0, 14.2. HRMS (ESI) *m/z* found: 601.2541, [M+H]^+^ C_34_H_37_N_2_O_8_ requires 601.2550.

#### 3.2.3. Magnesium(II) 1,4,8,11,15,18,22,25-octakis [4-(4-butoxycarbonylphenoxy)butyloxy]phthalocyanine (**3**)

Two alternative synthetic routes were elaborated:

i. Using **2** as a precursor

Magnesium turnings (4 mg, 0.133 mmol) were stirred in refluxing butan-1-ol with a catalytic amount of iodine for 3 h. After cooling, **2** (159 mg, 0.265 mmol) was added, and the reaction mixture was stirred under reflux for another 20 h under an inert gas atmosphere. When the reaction was completed, the mixture was cooled to room temperature and the solvent was evaporated. The residue was washed with a mixture of water and methanol (1:1). After drying in a vacuum, the solid was dissolved in DCM and filtered. The filtrate was collected, and the solvent evaporated. The obtained product was purified using column chromatography with silica gel and eluents DCM, DCM/MeOH 50:1, DCM/MeOH 35:1, DCM/MeOH 20:1, as well as C_18_-reversed-phase silica gel and eluents H_2_O/MeOH 3:1, DCM/MeOH 1:3, DCM to give 17 mg of green/yellow film **3** (2.4% yield).

ii. Using **8** as a precursor

Magnesium turnings (4 mg, 0.133 mmol) were stirred in refluxing butan-1-ol with a catalytic amount of iodine for 3 h. After cooling to room temperature, **8** (200 mg, 0.305 mmol) was added and the reaction mixture was stirred under reflux for another 20 h in an inert gas atmosphere. Next, the reaction mixture was cooled to room temperature and the solvent was evaporated. The residue was washed with a mixture of water and methanol (1:1). After drying in a vacuum, the solid was dissolved in DCM and filtered. The filtrate was collected and the solvent evaporated. The obtained product was purified using column chromatography with silica gel and eluents DCM, DCM/MeOH 50:1, DCM/MeOH 35:1, DCM/MeOH 20:1; C_18_-reversed-phase silica gel and eluents H_2_O/MeOH 3:1, DCM/MeOH 1:3, DCM to give 68 mg of green/yellow film **3** (34% yield).

R*_f_* (DCM/methanol 20:1) 0.31. UV-Vis (DCM): *λ_max_*, nm (log *ε*) 327 (4.99), 761 (5.06), 807 (5.23). ^1^H NMR (400 MHz, Pyr-*d*_5_) δ 8.09 (d, *J* = 9.0 Hz, 16H), 7.90 (s, 8H), 7.04 (d, *J* = 9.0 Hz, 16H), 5.24 (t, *J* = 6.5 Hz, 16H), 4.29 (d, *J* = 6.5 Hz, 16H), 4.26 (t, *J* = 6.5 Hz, 16H), 2.52 (m, 16H), 2.31 (m, 16H), 1.60 (m, 16H), 1.34 (m, 16H), 0.85 (t, *J* = 7.5 Hz, 24H). ^13^C NMR (101 MHz, Pyr-*d*_5_) δ 166.7, 163.8, 153.8, 152.6, 132.3, 130.4, 123.6, 119.9, 115.1, 73.0, 68.9, 65.0, 31.5, 27.3, 27.1, 19.9, 14.3. MS (MALDI) *m/z* found: 2649.2179, [M]^+^ C_152_H_176_MgN_8_O_32_ requires 2649.2241; 2650.2360, [M+H]^+^ C_152_H_177_MgN_8_O_32_ requires 2650.2319. HPLC purity (see Supplementary Materials).

#### 3.2.4. 2,3-. Bis[4-(4-ethoxycarbonylphenoxy)butylsulfanyl]but-2-enedinitrile (**4**)

Compound **1** (4.00 g, 13.28 mmol), dimercaptomaleonitrile sodium salt hydrate (0.99 g, 5.31 mmol), and potassium carbonate (7.34 g, 53.10 mmol) in 45 mL of anhydrous DMF were stirred at 50 °C for 20 h. Then, the reaction mixture was filtered through Celite and washed with DCM. The filtrate was evaporated and the crude residue was chromatographed on a silica gel (DCM, next DCM:MeOH 100:1) to give 1.532 g of a dense yellow liquid **4** (49% yield). R*_f_* (DCM/MeOH 50:1) 0.58. UV-Vis (DCM): *λ_max_*, nm (log *ε*) 343 (3.86), 258 (4.51). ^1^H NMR (500 MHz, DMSO-*d*_6_) δ 7.89 (d, *J* = 9.0 Hz, 4H), 6.99 (d, *J* = 9.0 Hz, 4H), 4.26 (q, *J* = 7.0 Hz, 4H), 4.05 (t, *J* = 5.5 Hz, 4H), 3.23 (t, *J* = 6.5 Hz, 4H), 1.82 (m, 8H), 1.29 (t, *J* = 7.0 Hz, 6H). ^13^C NMR (126 MHz, DMSO-*d*_6_) δ 165.3, 162.3, 131.1, 122.0, 121.1, 114.3, 112.3, 67.0, 60.2, 34.1, 27.1, 26.1, 14.1. HRMS (ESI) *m/z* found: 583.1927, [M+H]^+^ C_30_H_35_N_2_O_6_S_2_ requires 583.1937.

#### 3.2.5. Butyl 4-(4-bromobutoxy)benzoate (**5**)

Butyl 4-hydroxybenzoate (8.0 g, 41 mmol) and 1,4-dibromobutane (13.34 g, 7.26 mL, 62 mmol) were stirred with K_2_CO_3_ (28.5 g, 206 mmol) in 100 mL DMF at room temperature for 24 h. After that, solvents were evaporated and an oily residue was dissolved in DCM and hexane 1:1 mixture. The solution was filtered and the filtrate was evaporated under reduced pressure. Next, the residue was dissolved in DCM and washed with 10% NaOH and water. The collected organic phases were dried with MgSO_4_, evaporated to give a liquid residue, and chromatographed (silica gel, eluents: hexane, DCM/hexane 1:1) to yield 3.42 g of **5** (25% yield). R*_f_* (DCM/hexane 1:1) 0.21. UV-Vis (DCM): *λ_max_*, nm (log *ε*) 255 (4.69). ^1^H NMR (500 MHz, Pyr-*d*_5_) δ 8.18 (m, 2H, ArH, C2(C6)), 7.16 (m, 2H, ArH, C3(C5)), 5.51 (t, *J* = 7.5, 2H, BrCH_2_), 4.33 (t, *J* = 6.5, 2H, COOCH_2_), 4.17 (t, *J* = 6.5, 2H, BrCH_2_CH_2_CH_2_CH_2_), 2.41 (m, 2H, BrCH_2_CH_2_), 2.04 (m, 2H, BrCH_2_CH_2_CH_2_), 1.64 (m, 2H, CH_2_CH_2_CH_3_), 1.35 (m, 2H, CH_2_CH_3_), 0.86 (t, *J* = 7.5, 3H, CH_3_). ^13^C NMR (126 MHz, Pyr-*d*_5_) δ 166.8, 163.6, 132.4, 123.8, 115.4, 68.4, 65.1, 61.3, 31.5, 29.4, 26.5, 20.0, 14.3. MS ESI *m/z*: found 351.0575, calculated for C_15_H_21_BrO_3_Na [M+Na]^+^ 351.0572.

An additional byproduct was formed in this reaction, which readily precipitated upon addition of hexane to dichloromethane solution of the oily residue after DMF evaporation. This byproduct was obtained in the form of white powder in the amount of 3.9 g, and was identified as 1,4-bis(4-butylcarbonylphenyloxy)butane **5D** (43% yield).

R*_f_* (DCM/hexane 1:1) 0.05. UV-Vis (DCM): *λ_max_*, nm (log *ε*) 256 (4.40). ^1^H NMR (400 MHz, DMSO) δ 7.85 (m, 4H, ArH C2(C6)), 7.00 (m, 4H, ArH C3(C5)), 4.20 (t, *J* = 6.5, 4H, COOCH_2_), 4.09 (bs, 4H, ArOCH_2_), 1.87 (bs, 4H, ArOCH_2_CH_2_), 1.64 (m, 4H, COOCH_2_CH_2_CH_2_), 1.37 (m, 4H, COOCH_2_CH_2_), 0.90 (t, *J* = 7.5, 6H, CH_3_). ^13^C NMR (101 MHz, DMSO) δ 165.5, 162.5, 131.2, 122.0, 114.5, 67.5, 64.0, 30.3, 25.2, 18.8, 13.6. MS ESI *m/z*: found 465.2259, calculated for C_26_H_34_O_6_Na [M+Na]^+^ 465.2253.

#### 3.2.6. 2,3-Bis[4-(4-butoxycarbonylphenoxy)butylsulfanyl]but-2-enedinitrile (**6**)

Compound **5** (3.42 g, 10.38 mmol), dimercaptomaleonitrile sodium salt hydrate (0.77 g, 4.15 mmol), and potassium carbonate (5.74 g, 41.53 mmol) in 45 mL of anhydrous DMF were stirred at 50 °C for 20 h. Next, the reaction mixture was filtered through Celite and washed with DCM. The filtrate was evaporated and the residue was chromatographed (using silica gel and eluents: DCM, DCM/hexane 1:1, DCM/MeOH 100:1, DCM/MeOH 50:1) to give 1.24 g of yellow liquid **6** (yield 47%).

R*_f_* (DCM) = 0.12. UV-Vis (DCM): *λ_max_*, nm (log *ε*): 405 (4.87), 290 (5.21). ^1^H NMR (500 MHz, DMSO-*d*_6_) δ 7.88 (m, 4H, ArH C3(C5)), 7.00 (m, 4H, ArH C2(C6)), 4.22 (t, *J* = 6.5, 4H, COOCH_2_), 4.07 (t, *J* = 5.5, 4H, ArOCH_2_), 3.25 (t, *J* = 6.5, 4H, SCH_2_), 1.83 (m, 8H, SCH_2_CH_2_CH_2_), 1.67 (m, 4H, COOCH_2_CH_2_), 1.40 (m, 4H, COOCH_2_CH_2_CH_2_), 0.92 (t, *J* = 7.5, 6H, CH_3_). ^13^C NMR (126 MHz, DMSO-*d*_6_) δ 165.4, 162.3, 131.0, 122.0, 121.2, 114.3, 112.4, 67.1, 63.9, 34.1, 30.3, 27.1, 26.1, 18.7, 13.6. HRMS ESI *m/z* found: 661.2381, calculated for [M+Na]^+^ C_34_H_42_N_2_O_6_S_2_Na 661.2382.

#### 3.2.7. 2,3,7,8,12,13,17,18-Octakis[4-(4-butoxycarbonylphenoxy)butylsulfanyl]porphyrazinato magnesium(II) (**7**)

Magnesium turnings (6 mg, 0.235 mmol) were stirred in refluxing butan-1-ol with a catalytic amount of iodine for 3 h. After cooling, **6** (300 mg, 0.470 mmol) was added and the reaction mixture was stirred under reflux for another 20 h in an inert gas atmosphere. After cooling to room temperature, the solvent was evaporated. The solid residue was purified by column chromatography using silica gel and eluents DCM, DCM/MeOH 50:1; C_18_ reversed-phase silica gel and eluents H_2_O/MeOH 100:1, H_2_O/MeOH 35:1, H_2_O/MeOH 3:1, DCM/MeOH 1:3, DCM to give 98 mg of deep blue film **7** (32% yield).

R*_f_* (DCM/MeOH 50:1) 0.12. UV-Vis (DCM): *λ_max_*, nm (log *ε*): 672 (5.37), 376 (5.34), 255 (5.70). ^1^H NMR (500 MHz, DMSO-*d*_6_) δ 7.87 (m, 16H, ArH C3(C5)), 6.99 (m, 16H, ArH C2(C6)), 4.21 (t, *J* = 6.5, 16H, COOCH_2_), 4.06 (t, *J* = 5.5, 16H, ArOCH_2_), 3.24 (t, *J* = 6.5, 16H, SCH_2_CH_2_CH_2_), 1.83 (m, 32 H, SCH_2_CH_2_CH_2_), 1.66 (m, 16H, COOCH_2_CH_2_), 1.39 (m, 16 H, COOCH_2_CH_2_CH_2_), 0.92 (t, *J* = 7.5, 24H, CH_3_). ^13^C NMR (126 MHz, DMSO-*d*_6_) δ 165.4, 162.3, 131.0, 122.0, 121.2, 114.3, 112.4, 67.1, 63.9, 34.1, 30.3, 27.1, 26.1, 18.7, 13.6. HRMS ESI *m/z* found: 2465.7714, calculated for [M+H]^+^ C_128_H_153_MgN_8_O_24_S_8_ 2465.8614. HPLC purity (see Supplementary Materials).

#### 3.2.8. 1,2-Dicyano-3,6-bis[4-(4-butoxycarbonylphenoxy)butyloxy]benzene (**8**)

3,6-Dihydroxybenzene-1,2-dicarbonitrile (389 mg, 2.43 mmol) and **5** (2.00 g, 6.07 mmol) were stirred with K_2_CO_3_ (1.68 g, 12.15 mmol) in 40 mL DMF at room temperature for 72 h. After that, the reaction mixture was poured on ice (50 mL) and filtered. The solid was washed with water and methanol to yield 1.21 g of white solid **8** (yield 76%).

R*_f_* (DCM/MeOH 50:1) 0.64. UV-Vis (DCM): *λ_max_*, nm (log *ε*): 356 (3.87), 251 (4.74), 228 (4.63). ^1^H NMR (700 MHz, DMSO-*d*_6_) δ 7.85 (m, 4H, ArH C3(C5)), 7.59 (s, 2H, hydroquinone ArH), 7.00 (m, 4H, ArH C2(C6)), 4.20 (m, 8H, COOCH_2_ and OCH_2_CH_2_CH_2_CH_2_), 4.12 (s, 4H, OCH_2_CH_2_CH_2_CH_2_), 1.89 (s, 8H, OCH_2_CH_2_CH_2_CH_2_), 1.65 (m, 4H, COOCH_2_CH_2_), 1.38 (m, 4H,CH_2_CH_3_), 0.90 (t, *J* = 7.5, 6H, CH_3_). ^13^C NMR (176 MHz, DMSO-*d*_6_) δ 165.4, 162.4, 154.8, 131.2, 122.0, 120.5, 114.4, 113.6, 102.8, 69.4, 67.4, 64.0, 30.3, 25.0, 18.8, 13.6. MS ESI *m/z*: found 679.2988, calculated for C_38_H_44_N_2_O_8_Na [M+Na]^+^ 679.2995.

### 3.3. Photochemical Studies

Singlet oxygen formation quantum yields in solution were measured according to the procedure described before [48,59,60,66]. 1,3-Diphenylisobenzofuran (DPBF) was used as a singlet oxygen chemical quencher, whereas ZnPc was applied as a reference Φ_∆_ generator. The photostability of studied macrocycles in solution was also evaluated and determined using the photodecomposition quantum yields (Φ_P_) [60,66,67,68]. Fluorescence quantum yields were determined with the earlier described indirect method, with the reference compound–ZnPc [48,59,61,69].

### 3.4. Deposition of 3 and 7 on TiO_2_ P25 Nanoparticles

Obtained macrocyclic compounds were deposited on P25 Aeroxide^®^ (Merck KGaA, Darmstadt, Germany) TiO_2_ nanoparticles using the chemical deposition method [70]. Briefly, to a dispersion of P25 nanoparticles (sized ~21 nm) in dichloromethane (100 mg in 20 mL), a solution of either **3** or **7** (10 mg) in dichloromethane (5 mL) was added, and the reaction mixture was stirred for 16 h. Next, the solvent was evaporated under reduced pressure and the material was dried in air. The ratio of the macrocycle to the P25 was assessed to be 1:10 (*w*/*w*).

The nanoparticles were characterized by particle size measurements. The nanoparticle size distribution was analyzed using Malvern Panalytical NanoSight LM10 instrument (sCMOS camera, 405 nm laser; Malvern, United Kingdom) using NTA (Nanoparticle Tracking Analysis) 3.2 Dev Build 3.2.16 software (Malvern, United Kingdom). Before the measurements, the dispersions of the materials were diluted with water to achieve the operating range of nanoparticle concentration. The temperature of the sample chamber was set and maintained at 25.0 ± 0.1 °C, and the syringe pump infusion rate was set to 200.

### 3.5. Acute Toxicity Assessment

The toxicity of materials was assessed using the Microtox^®^ (Modern Water, Cambridge, UK) acute toxicity test [63]. The test was modified to assess compounds’ phototoxic potential, following the previously presented methodology [18]. Briefly, two sets of experiments were conducted: dark toxicity and light toxicity tests. An additional red-light diode (λ_max_ = 665 nm) was mounted above the Microtox^®^ M500 (Modern Water, Cambridge, UK) cuvette incubator to study compounds and materials’ photocytotoxicities. The red-light diode was used to irradiate the prepared samples upon combining with *Aliivibrio fischeri* bacteria. The light intensity was set to 7.0 mW/cm^2^ at the samples’ surface (as measured with Radiometer RD 0.2/2 with TD probe, Optel). Cell viability was calculated using Modern Water MicrotoxOmni 4.2 software (Modern Water, London, UK), according to bioluminescence emitted by the bacteria measured with Microtox^®^ M500. Macrocycles **3** and **7** were solubilized with DMSO (1% v/v). Distilled water was used as the solvent for all the materials. The concentrations of the porphyrinoids added to *A. fischeri* cultures were 10^−4^ mol/dm^3^.

## 4. Conclusions

Two new macrocycles were synthesized, namely magnesium(II) 1,4,8,11,15,18,22,25-octakis[4-(4-butoxycarbonylphenoxy)butyloxy]phthalocyanine (**3**) and 2,3,7,8,12,13,17,18-octakis[4-(4-butoxycarbonylphenoxy)butylsulfanyl]porphyrazinato magnesium(II) (**7**). The synthesis of both compounds was optimized by changing the starting material from ethyl 4-hydroxybenzoate to 1-butyl 4-hydroxybenzoate, which resulted in higher yields of Pc **3** synthesis and allowed us to synthesize and isolate Pz **7**. Both macrocycles were characterized using spectral methods—NMR (^1^H NMR, ^13^C NMR, ^1^H-^1^H COSY, ^1^H-^13^C HSQC, ^1^H-^13^C HMBC), UV-Vis spectroscopy, and mass spectrometry. Each macrocycle revealed high photostability (Φ_P_ ~ 10^−6^). However, when it comes to emission properties, only Pc **3** revealed fluorescence, whereas Pz **7** did not exhibit emission. In addition, Pc **3** also exhibited much higher quantum yields of singlet oxygen generation, which reached 0.29 in DMF and 0.13 in DMSO, in comparison to Pz **7**, with 0.02 in DMF and 0.09 in DMSO.

After depositing **3** and **7** on P25 titania nanoparticles, the obtained nanoparticles **3**@P25 and **7**@P25 were studied in a Microtox^®^ screening test. In the case of both materials tested, dark toxicity reached almost 80%, but the effect was the strongest together with irradiation, reaching nearly a 100% decrease in cell viability. It was found that **3**@P25 nanoparticles, in comparison to **3** alone, revealed a higher cytotoxicity/photocytotoxicity. Further potential of this material as a photocatalyst for decontamination of microbially polluted water could be considered.

## Figures and Tables

**Table 1:** The particle size distribution of P25, **3**@P25 and **7**@P25.

Material	Measured Particle Size [nm]	Polydispersity Index ^a^
P25	132.1 ± 85.1	0.42
**3**@P25	148.4 ± 99.3	0.45
**7**@P25	159.1 ± 101.2	0.40

^a^ calculated according to the formula PDI = (SD/mean diameter)^2^ [39].

## Data Availability

The data presented in this study are available on request from the corresponding author.

## Supplementary Materials

The following are available online, Table S1: ^1^H and ^13^C NMR data obtained for **1** including key correlations determined from ^1^H-^1^H COSY, ^1^H-^13^C HSQC and ^1^H-^13^C HMBC spectra. Figure S1: ^1^H and (^13^C) chemical shift values [ppm] and key correlations observed in NMR spectra of **1**. Bold lines: ^1^H-^1^H COSY; Arrows: ^1^H-^13^C HMBC. Figure S2: ^1^H NMR spectrum of **1** (400 MHz, DMSO-*d_6_*, 298 K). The symbols * and ~ indicate DMSO-*d_6_* and water residual peaks, respectively. Figure S3: ^13^C NMR spectrum recorded for **1** (101 MHz, DMSO-*d_6_*, 298 K). The symbol * indicates DMSO-*d_6_* residual peak. Table S2: ^1^H and ^13^C NMR data obtained for **2** including key correlations determined from ^1^H-^1^H COSY, ^1^H-^13^C HSQC and ^1^H-^13^C HMBC spectra. Figure S4:  ^1^H and (^13^C) chemical shift values [ppm] and key correlations observed in NMR spectra of **2**. Bold lines: ^1^H-^1^H COSY; Arrows: ^1^H-^13^C HMBC. Figure S5: ^1^H NMR spectrum of **2** (500 MHz, DMSO-*d_6_*, 298 K). The symbols * and ~ indicate DMSO-*d_6_* and water residual peaks, respectively. Figure S6: ^13^C NMR spectrum recorded for **2** (126 MHz, DMSO-*d_6_*, 298 K). The symbol * indicates DMSO-*d_6_* residual peak. Table S3: ^1^H and ^13^C NMR data obtained for **3** including key correlations determined from ^1^H-^1^H COSY, ^1^H-^13^C HSQC and ^1^H-^13^C HMBC spectra. Figure S7: ^1^H and (^13^C) chemical shift values [ppm] and key correlations observed in NMR spectra of **3**. Bold lines: ^1^H-^1^H COSY; Arrows: ^1^H-^13^C HMBC. Figure S8: ^1^H NMR spectrum of **3** (500 MHz, pyridine-*d_5_*, 298 K). The symbols # and ~ indicate pyridine-*d_5_* and water residual peaks, respectively. Figure S9: ^13^C NMR spectrum recorded for **3** (126 MHz, pyridine-*d_5_*, 298 K). The symbols # indicate pyridine-*d_5_* residual peaks. Table S4: ^1^H and ^13^C NMR data obtained for **4** including key correlations determined from ^1^H-^1^H COSY, ^1^H-^13^C HSQC and ^1^H-^13^C HMBC spectra. Figure S10: ^1^H and (^13^C) chemical shift values [ppm] and key correlations observed in NMR spectra of **4**. Bold lines: ^1^H-^1^H COSY; Arrows: ^1^H-^13^C HMBC. Figure S11: ^1^H NMR spectrum of **4** (400 MHz, DMSO-*d_6_*, 298 K). The symbols * and ~ indicate DMSO-*d_6_* and water residual peaks, respectively. Figure S12: ^13^C NMR spectrum recorded for **4** (101 MHz, DMSO-*d_6_*, 298 K). The symbol * indicates DMSO-*d_6_* residual peak. **Figure S13**. Presumed structure of **4** macrocyclization product. Table S5: ^1^H and ^13^C NMR data obtained for the product of **4** macrocyclization including key correlations determined from ^1^H-^1^H COSY, ^1^H-^13^C HSQC and ^1^H-^13^C HMBC spectra. Figure S14: ^1^H and (^13^C) chemical shift values [ppm] and key correlations observed in NMR spectra of the product of **4** macrocyclization. Bold lines: ^1^H-^1^H COSY; Arrows: ^1^H-^13^C HMBC. Figure S15: ^1^H NMR spectrum of the product of **4** macrocyclization (500 MHz, pyridine-*d_5_*, 298 K). The symbols # and ~ indicate pyridine-*d_5_* and water residual peaks, respectively. Figure S16: ^13^C NMR spectrum recorded for the product of **4** macrocyclization (126 MHz, pyridine-*d*_5_, 298 K). The symbol # indicates pyridine-*d*_5_ residual peaks. Table S6: ^1^H and ^13^C NMR data obtained for **5** including key correlations determined from ^1^H-^1^H COSY, ^1^H-^13^C HSQC and ^1^H-^13^C HMBC spectra. Figure S17: ^1^H and (^13^C) chemical shift values [ppm] and key correlations observed in NMR spectra of **5**. Bold lines: ^1^H-^1^H COSY; Arrows: ^1^H-^13^C HMBC. Figure S18: ^1^H NMR spectrum of **5** (500 MHz, pyridine-*d_5_*, 298 K). The symbols * and ~ indicate pyridine-*d_5_* and water residual peaks, respectively. Figure S19: ^13^C NMR spectrum recorded for **5** (126 MHz, pyridine-*d_5_*, 298 K). The symbol * indicates pyridine-*d_5_* residual peak. Table S7: ^1^H and ^13^C NMR data obtained for **5D** including key correlations determined from ^1^H-^1^H COSY, ^1^H-^13^C HSQC and ^1^H-^13^C HMBC spectra. Figure S20: ^1^H and (^13^C) chemical shift values [ppm] and key correlations observed in NMR spectra of **5D**. Bold lines: ^1^H-^1^H COSY; Arrows: ^1^H-^13^C HMBC. Figure S21: ^1^H NMR spectrum of **5D** (400 MHz, DMSO-*d_6_*, 298 K). The symbols # and ~ indicate DMSO-*d_6_* and water residual peaks, respectively. Figure S22: ^13^C NMR spectrum recorded for **5D** (101 MHz, DMSO-*d_6_*, 298 K). The symbol # indicates DMSO-*d_6_* residual peak. Table S8: ^1^H and ^13^C NMR data obtained for **6** including key correlations determined from ^1^H-^1^H COSY, ^1^H^13^C HSQC and ^1^H-^13^C HMBC spectra. Figure S23: ^1^H and (^13^C) chemical shift values [ppm] and key correlations observed in NMR spectra of **6**. Bold lines: ^1^H-^1^H COSY; Arrows: ^1^H-^13^C HMBC. Figure S24: ^1^H NMR spectrum of **6** (500 MHz, DMSO-*d_6_*, 298 K). The symbols # and ~ indicate DMSO-*d_6_* and water residual peaks, respectively. Figure S25: ^13^C NMR spectrum recorded for **6** (126 MHz, DMSO-*d_6_*, 298 K). The symbol # indicates DMSO-*d_6_* residual peak. Table S9: ^1^H and ^13^C NMR data obtained for **7** including key correlations determined from ^1^H-^1^H COSY, ^1^H-^13^C HSQC and ^1^H-^13^C HMBC spectra. Figure S26: ^1^H and (^13^C) chemical shift values [ppm] and key correlations observed in NMR spectra of **7**. Bold lines: ^1^H-^1^H COSY; Arrows: ^1^H-^13^C HMBC. Figure S27: ^1^H NMR spectrum of **7** (500 MHz, DMSO-*d_6_*, 298 K). The symbols # and ~ indicate DMSO-*d_6_* and water residual peaks, respectively. Figure S28: ^13^C NMR spectrum recorded for **7** (126 MHz, DMSO-*d_6_*, 298 K). The symbol # indicates DMSO-*d_6_* residual peak. Table S10: ^1^H and ^13^C NMR data obtained for **8** including key correlations determined from ^1^H-^1^H COSY, ^1^H-^13^C HSQC and ^1^H-^13^C HMBC spectra. Figure S29: ^1^H and (^13^C) chemical shift values [ppm] and key correlations observed in NMR spectra of **8**. Bold lines: ^1^H-^1^H COSY; Arrows: ^1^H-^13^C HMBC. Figure S30: ^1^H NMR spectrum of **8** (700 MHz, DMSO-*d_6_*, 298 K). The symbols # and ~ indicate DMSO-*d_6_* and water residual peaks, respectively. Figure S31: ^13^C NMR spectrum recorded for **8** (176 MHz, DMSO-*d_6_*, 298 K). The symbol # indicates DMSO-*d_6_* residual peak. Figure S32: Synthetic approaches towards A_3_B macrocycles: (i) 1,2-dicyanobenzene (tenfold excess), (BuO)_2_Mg, BuOH, reflux, 20 h.

## Sample Availability

Samples of the compounds **1**–**8** are available from the authors.
