# Supplementary material for: Nipagin-Functionalized Porphyrazine and Phthalocyanine—Synthesis, Physicochemical Characterization and Toxicity Study after Deposition on Titanium Dioxide Nanoparticles P25
Source: Molecules. 2021 May 1;26(9):2657. doi: 10.3390/molecules26092657 (PMC8124671; doi:10.3390/molecules26092657)
Supplement: Supplementary file 1 [file molecules-26-02657-s001.zip › molecules-1168740-supplementary.pdf]

Supplementary Materials for

Nipagin-functionalized porphyrazine and phthalocyanine - synthesis, physicochemical characterization and toxicity study after deposition on titanium dioxide nanoparticles P25

Dariusz T. Mlynarczyk [1,*], Daniel Ziental [2], Emil Kolasinski [1], Lukasz Sobotta [2],

Tomasz Koczorowski [1], Jadwiga Mielcarek [2], Tomasz Goslinski [1]

[1] *Chair and Department of Chemical Technology of Drugs, Poznan University of Medical Sciences, Grunwaldzka 6, 60-780 Poznań, Poland*

[2] *Chair and Department of Inorganic and Analytical Chemistry, Poznan University of Medical Sciences, Grunwaldzka 6, 60-780 Poznań, Poland*

*Correspondence to: D.T. Mlynarczyk, Chair and Department of Chemical Technology of Drugs, Poznan University of Medical Sciences, Grunwaldzka 6, 60-780 Poznań, Poland.
Tel.: (+48) 61 854 66 33, Fax: (+48) 61 854 66 39. E-mail: mlynarczykd@ump.edu.pl

TABLE OF CONTENTS

1. EXPERIMENTAL S3

1.1. NMR DATA OF **1** S3
1.2. NMR DATA OF **2** S5
1.3. NMR DATA OF **3** S7
1.4. NMR DATA OF **4** S9
1.5. NMR DATA OF **4** MACROCYCLIZATION PRODUCT – AN UNSYMMETRICAL PORPHYRAZINE S11
1.6. NMR DATA OF **5** S14
1.7. NMR DATA OF **5D** S16
1.8. NMR DATA OF **6** S18
1.9. NMR DATA OF **7** S20
1.10. NMR DATA OF **8** S22
1.11. HPLC DATA OF **3** S24
1.12. HPLC DATA OF **7** S27

2. SYNTHETIC ATTEMPTS TOWARDS A_3B TYPE MACROCYCLES. S30

1. Experimental

1.1. NMR data of 1

Table S1. ^{1}H and ^{13}C NMR data obtained for **1** including key correlations determined from ^{1}H–^{1}H COSY, ^{1}H–^{13}C HSQC and ^{1}H–^{13}C HMBC spectra.

δ_H (ppm)	Multiplicity ($J_{H–H}$ in Hz)	^{1}H–^{1}H COSY δ_H (ppm)	^{1}H–^{13}C HSQC δ_C (ppm)	^{1}H–^{13}C HMBC δ_C (ppm)		
7.90	d (9.0)	7.03	131.1	165.3<br>122.1	162.3<br>114.3	131.1
7.03	d (9.0)	7.90	114.3	165.3<br>122.1	162.3<br>114.3	131.1
4.25	q (7.0)	1.29	60.2	165.3<br>66.9	122.1<br>14.2	114.3
4.07	t (6.5)	1.84	66.9	162.3	29.0	27.2
3.59	t (6.5)	1.96	34.6	29.0	27.2	
1.96	m	3.59 1.84	29.0	66.9	34.6	27.2
1.84	m	4.07 1.96	27.2	66.9	34.6	29.0
1.29	t (7.0)	4.25	14.2	162.3	122.1	60.2

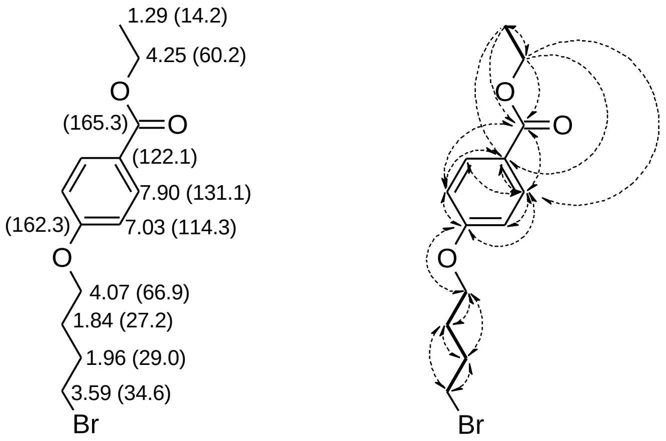

Fig. S1. ^{1}H and (^{13}C) chemical shift values [ppm] and key correlations observed in NMR spectra of **1**. Bold lines: ^{1}H–^{1}H COSY; Arrows: ^{1}H–^{13}C HMBC.

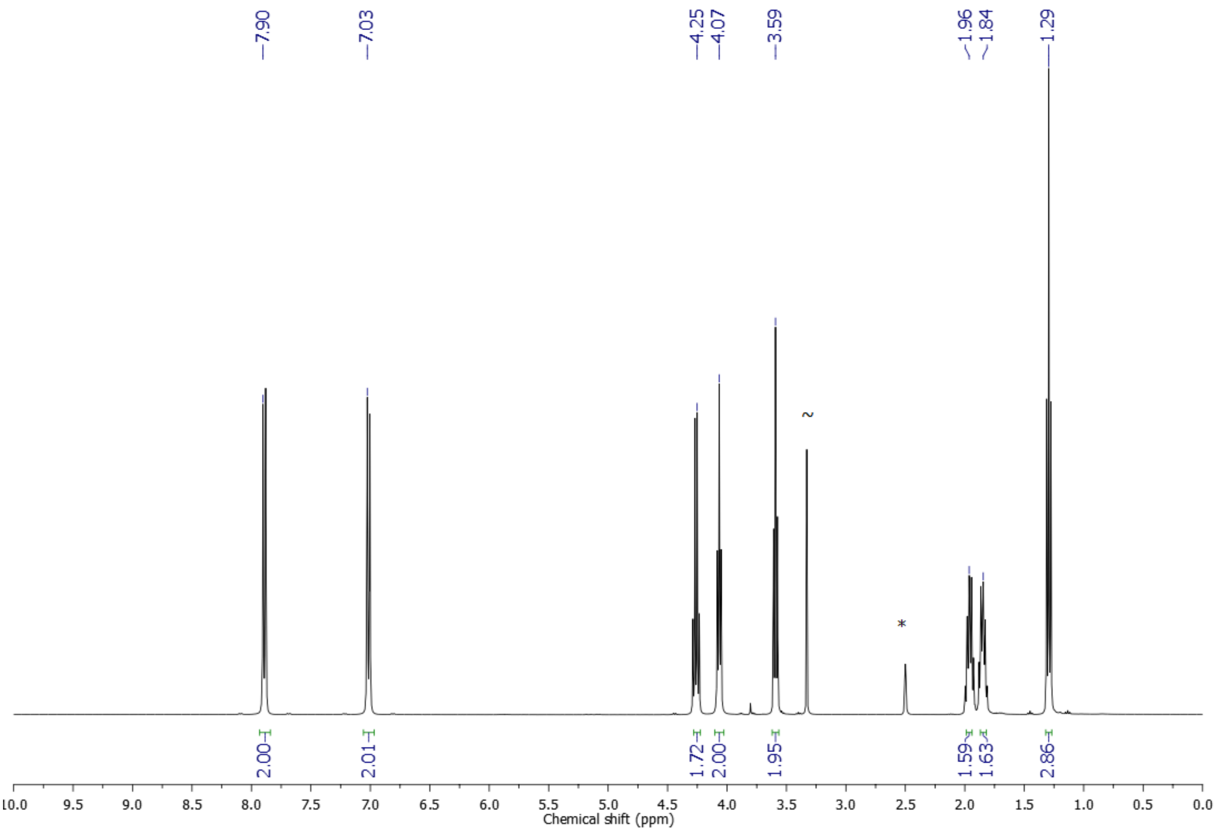

Fig. S2. ^{1}H NMR spectrum of **1** (400 MHz, DMSO-d_6, 298 K). The symbols * and ~ indicate DMSO-d_6 and water residual peaks, respectively.

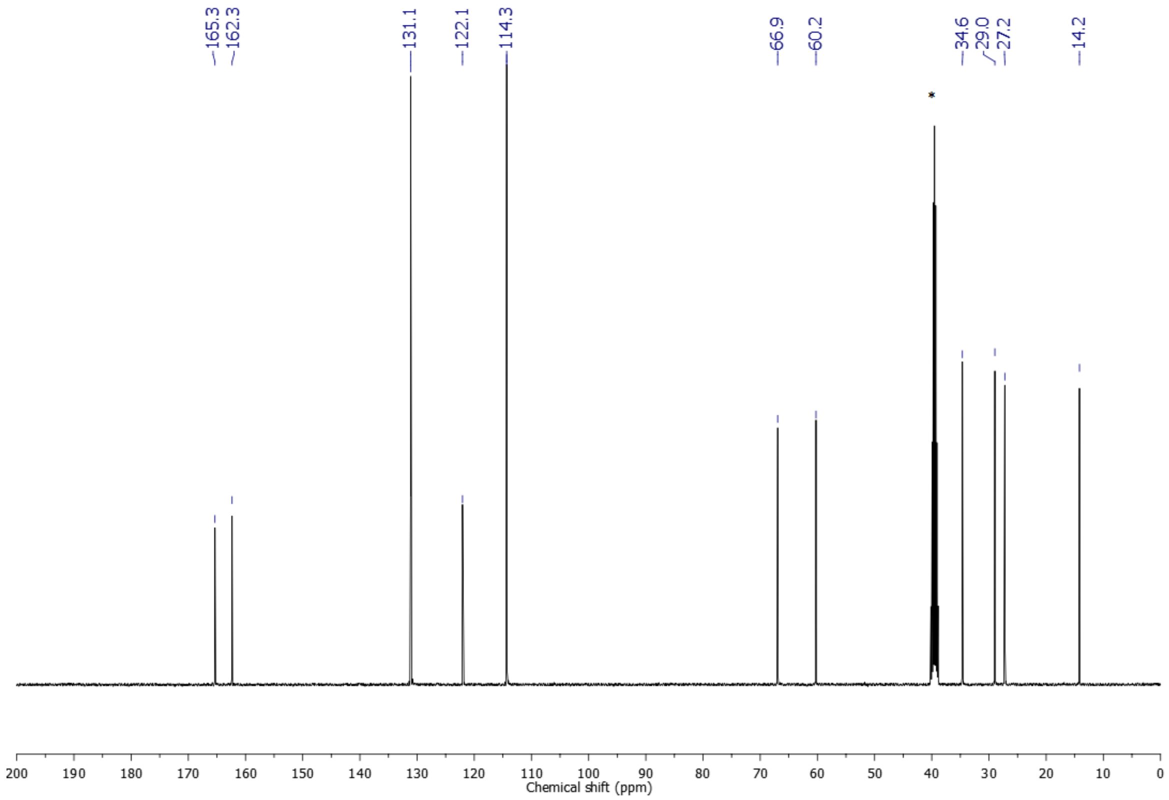

Fig. S3. ^{13}C NMR spectrum recorded for **1** (101 MHz, DMSO-d_6, 298 K). The symbol * indicates DMSO-d_6 residual peak.

1.2. NMR data of 2

Table S2. ^{1}H and ^{13}C NMR data obtained for **2** including key correlations determined from ^{1}H–^{1}H COSY, ^{1}H–^{13}C HSQC and ^{1}H–^{13}C HMBC spectra.

δ_H (ppm)	Multiplicity ($J_{H–H}$ in Hz)	^{1}H–^{1}H COSY δ_H (ppm)	^{1}H–^{13}C HSQC δ_C (ppm)	^{1}H–^{13}C HMBC δ_C (ppm)			
7.89	d (8.5)	7.02	131.1	165.4	162.4	131.1	114.4
7.61	s	-	120.5	154.8	113.5	102.8	
7.02	d (8.5)	7.89	114.4	162.4	122.0	114.4	
4.25	m	1.30	60.3	165.4	14.2		
4.23	m	1.91	69.5	154.8	25.0		
4.14	m	1.91	67.4	162.4	25.0		
1.91	m	4.23 4.14	25.0	69.5	67.4		
1.30	t (7.0)	4.25	14.2	60.3			

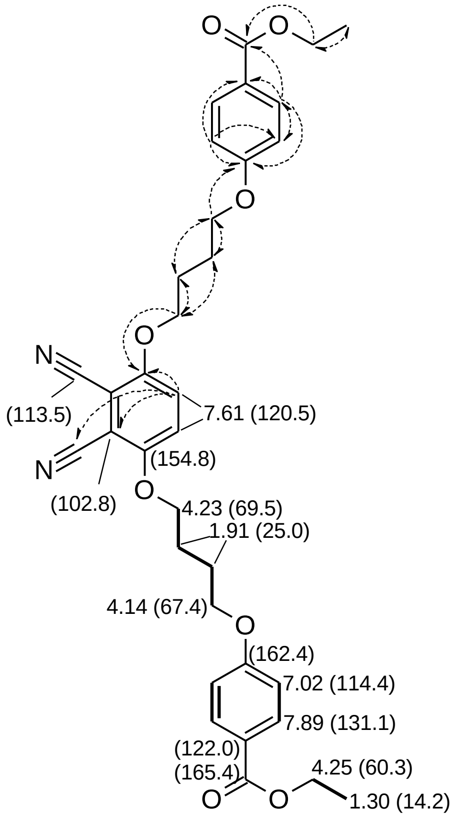

Fig. S4. ^{1}H and (^{13}C) chemical shift values [ppm] and key correlations observed in NMR spectra of **2**. Bold lines: ^{1}H–^{1}H COSY; Arrows: ^{1}H–^{13}C HMBC.

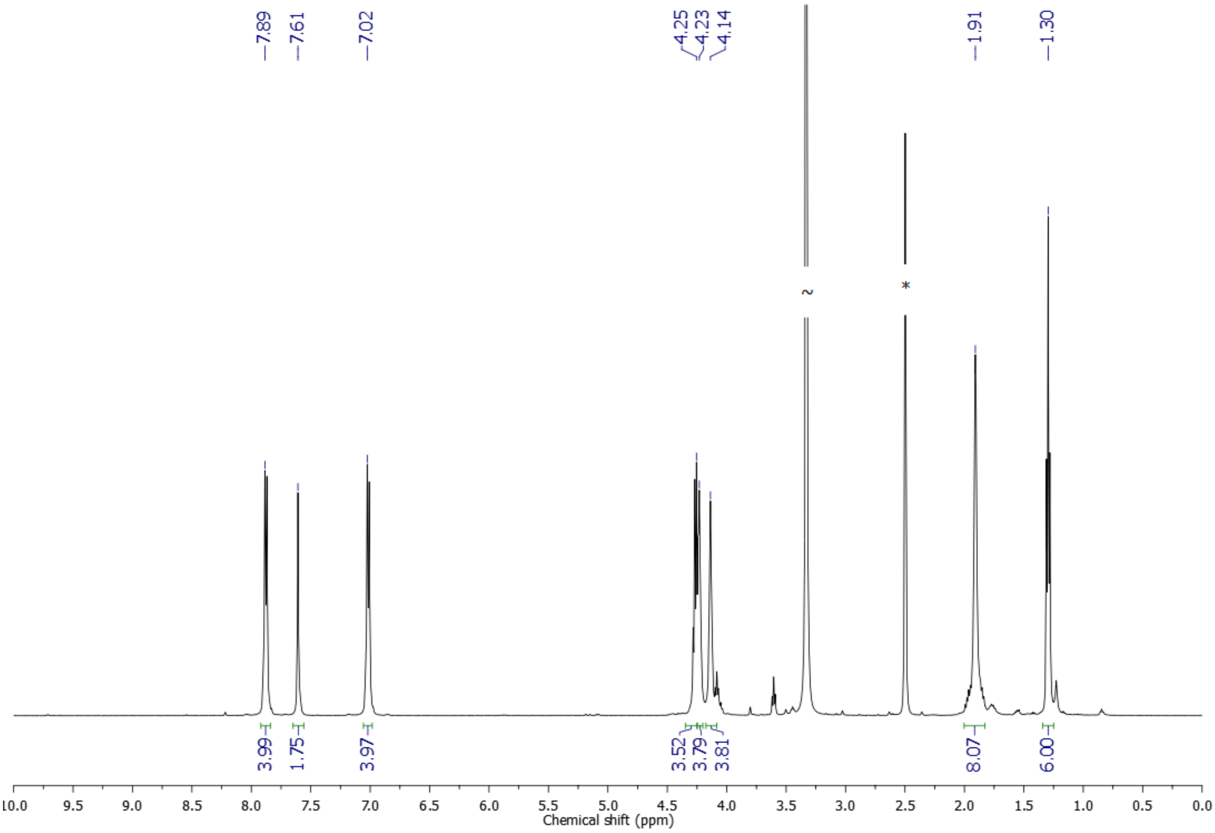

Fig. S5. ^{1}H NMR spectrum of **2** (500 MHz, DMSO-d_6, 298 K). The symbols * and ~ indicate DMSO-d_6 and water residual peaks, respectively.

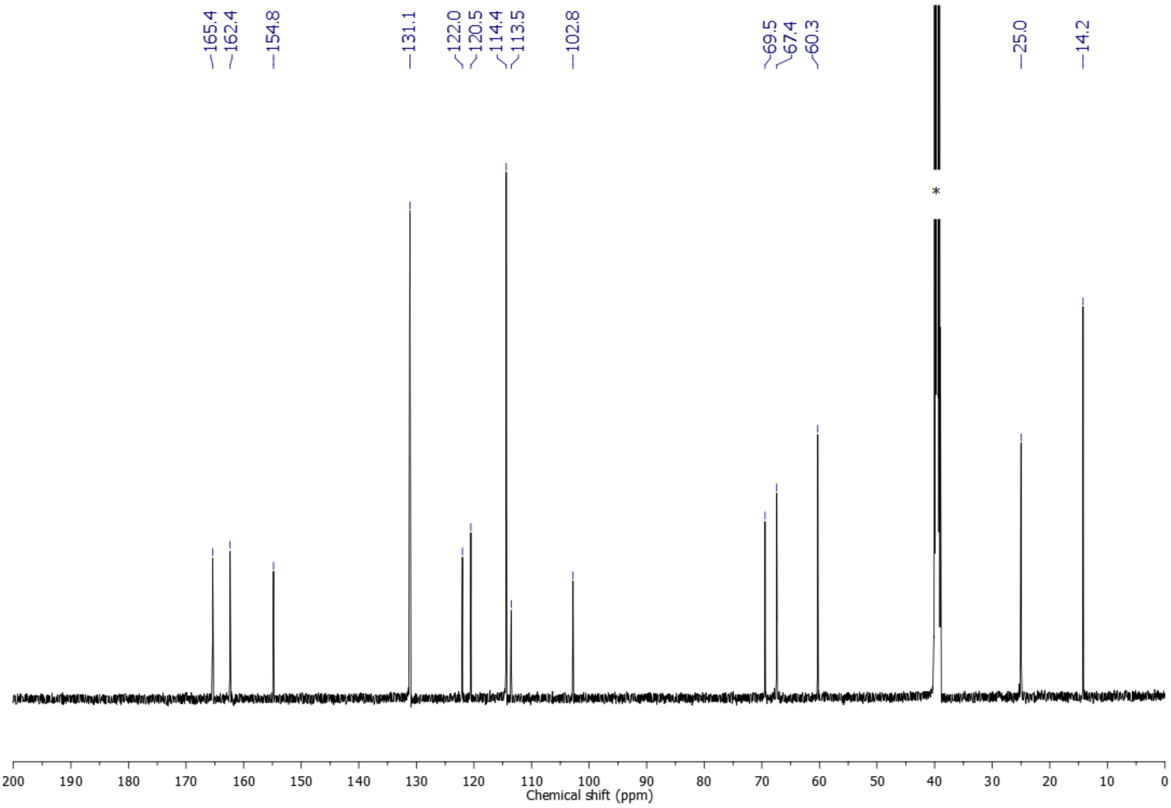

Fig. S6. ^{13}C NMR spectrum recorded for **2** (126 MHz, DMSO-d_6, 298 K). The symbol * indicates DMSO-d_6 residual peak.

1.3. NMR data of 3

Table S3. ^{1}H and ^{13}C NMR data obtained for **3** including key correlations determined from ^{1}H–^{1}H COSY, ^{1}H–^{13}C HSQC and ^{1}H–^{13}C HMBC spectra.

δ_H (ppm)	Multiplicity ($J_{H–H}$ in Hz)	^{1}H–^{1}H COSY δ_H (ppm)	^{1}H–^{13}C HSQC δ_C (ppm)	^{1}H–^{13}C HMBC δ_C (ppm)
8.09	d (9.0)	7.04	132.3	166.7 163.8 132.3 123.6 115.1
7.90	s	-	119.9	153.8 152.6 130.4
7.04	d (9.0)	8.09	115.1	166.7 163.8 123.6
5.24	t (6.5)	2.52	73.0	152.6 27.3
4.29	t (6.5)	1.60	65.0	166.7 132.3 19.9
4.26	t (6.5)	2.31	68.9	163.8 27.1
2.52	m	5.24 2.31	27.3	73.0 68.9 27.1
2.31	m	4.26 2.52	27.1	73.0 68.9 27.3
1.60	m	4.29 1.34	31.5	65.0 19.9 14.3
1.34	m	1.60 0.84	19.9	65.0 31.5 14.3
0.85	t (7.5)	1.34	14.3	31.5 19.9

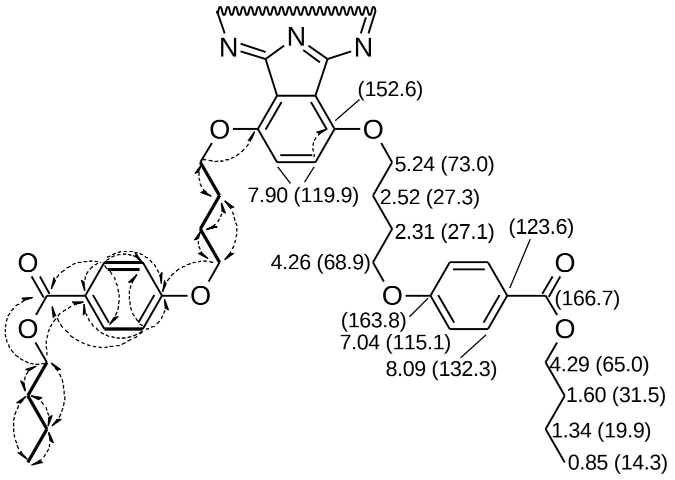

Fig. S7. ^{1}H and (^{13}C) chemical shift values [ppm] and key correlations observed in NMR spectra of **3**. Bold lines: ^{1}H–^{1}H COSY; Arrows: ^{1}H–^{13}C HMBC.

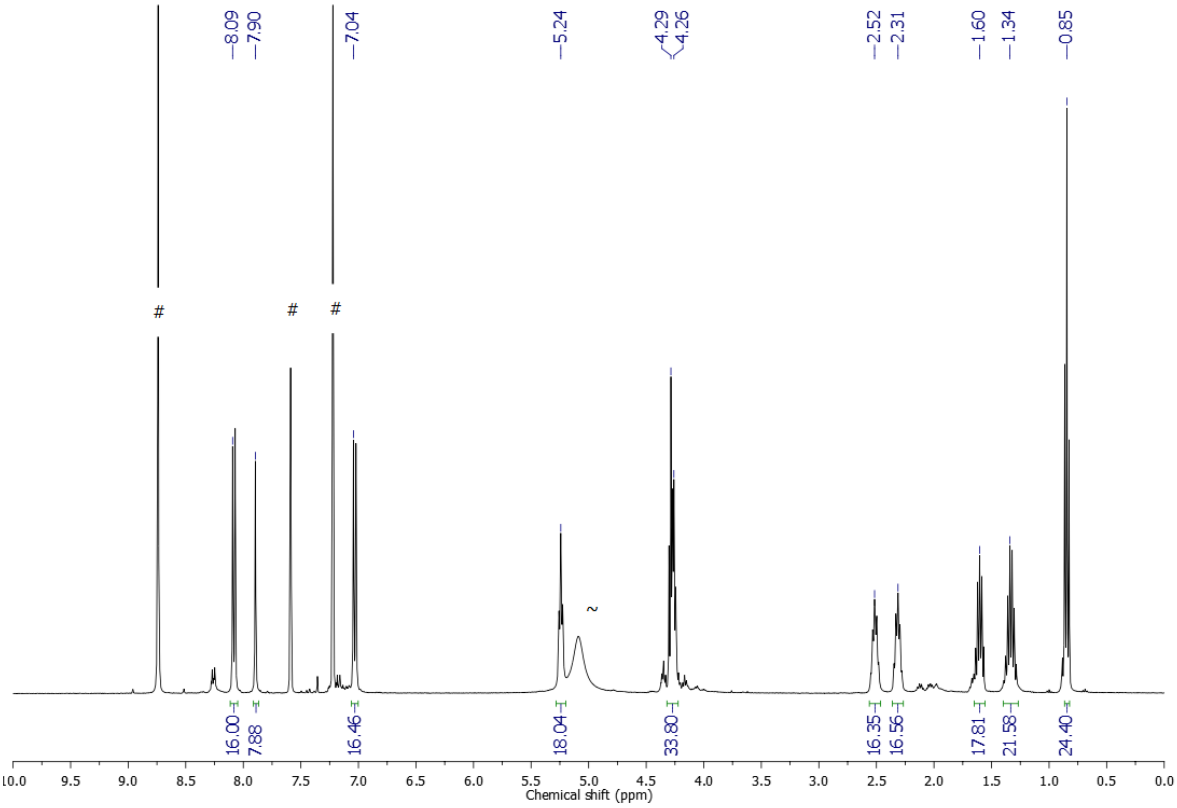

Fig. S8. ^{1}H NMR spectrum of **3** (500 MHz, pyridine-d_5, 298 K). The symbols # and ~ indicate pyridine-d_5 and water residual peaks, respectively.

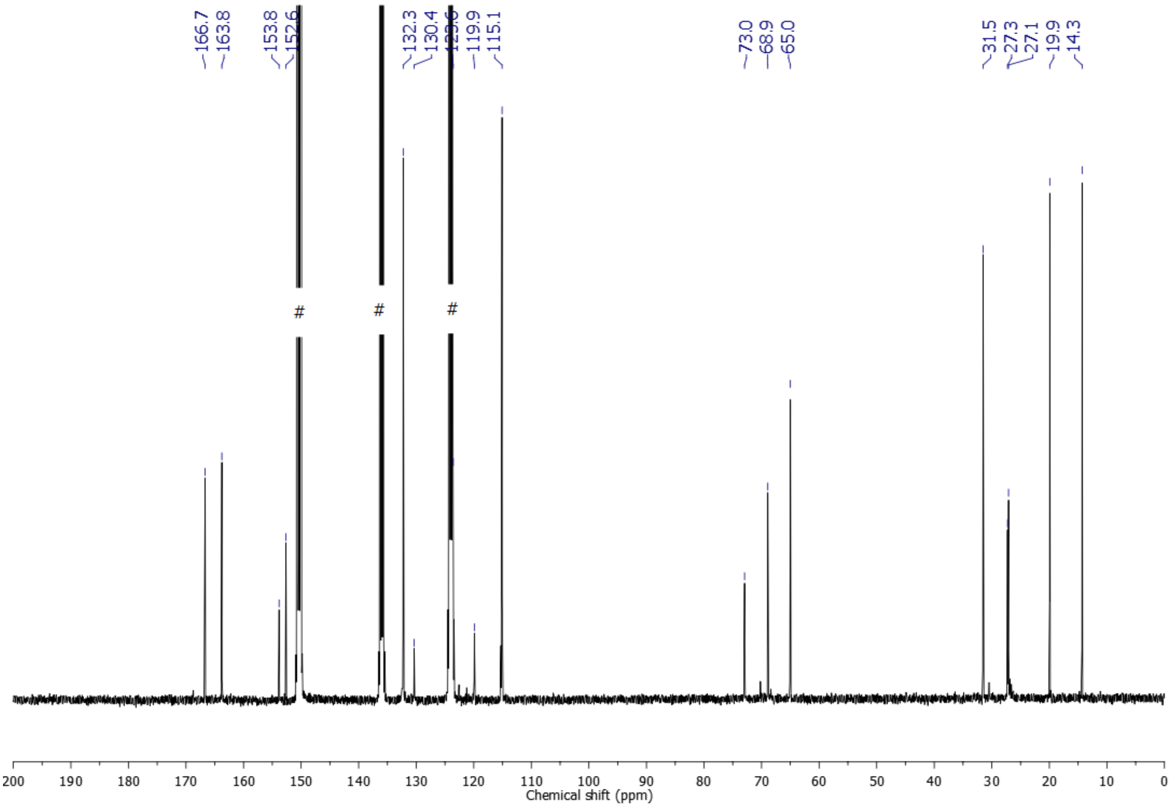

Fig. S9. ^{13}C NMR spectrum recorded for **3** (126 MHz, pyridine-d_5, 298 K). The symbols # indicate pyridine-d_5 residual peaks.

1.4. NMR data of 4

Table S4. ^{1}H and ^{13}C NMR data obtained for **4** including key correlations determined from ^{1}H–^{1}H COSY, ^{1}H–^{13}C HSQC and ^{1}H–^{13}C HMBC spectra.

δ_H (ppm)	Multiplicity ($J_{H–H}$ in Hz)	^{1}H–^{1}H COSY δ_H (ppm)	^{1}H–^{13}C HSQC δ_C (ppm)	^{1}H–^{13}C HMBC δ_C (ppm)			
7.89	d (9.0)	6.99	131.1	165.3 114.3	162.3	131.1	122.0
6.99	d (9.0)	7.89	114.3	165.3 114.3	162.3	131.1	122.0
4.26	q (7.0)	4.26 1.29	60.2	165.3	122.0	14.1	
4.05	t (5.5)	4.05 1.82	67.0	162.3	27.1	26.1	
3.23	t (6.5)	3.23 1.82	34.1	121.1	67.0	27.1	26.1
1.82	m	4.05 3.23 1.82	27.1 26.1	67.0	34.1	27.1	26.1
1.29	t (7.5)	4.26 1.29	14.1	60.2			
Other carbon atoms (ppm): 112.3.							

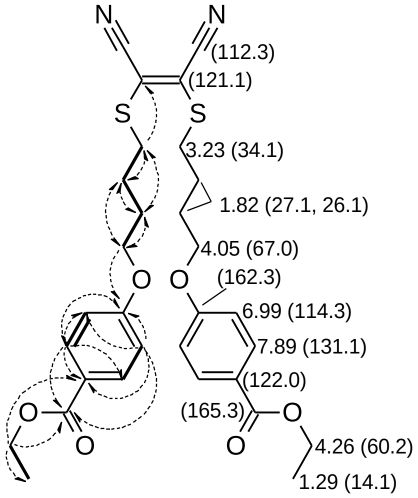

Fig. S10. ^{1}H and (^{13}C) chemical shift values [ppm] and key correlations observed in NMR spectra of **4**. Bold lines: ^{1}H–^{1}H COSY; Arrows: ^{1}H–^{13}C HMBC.

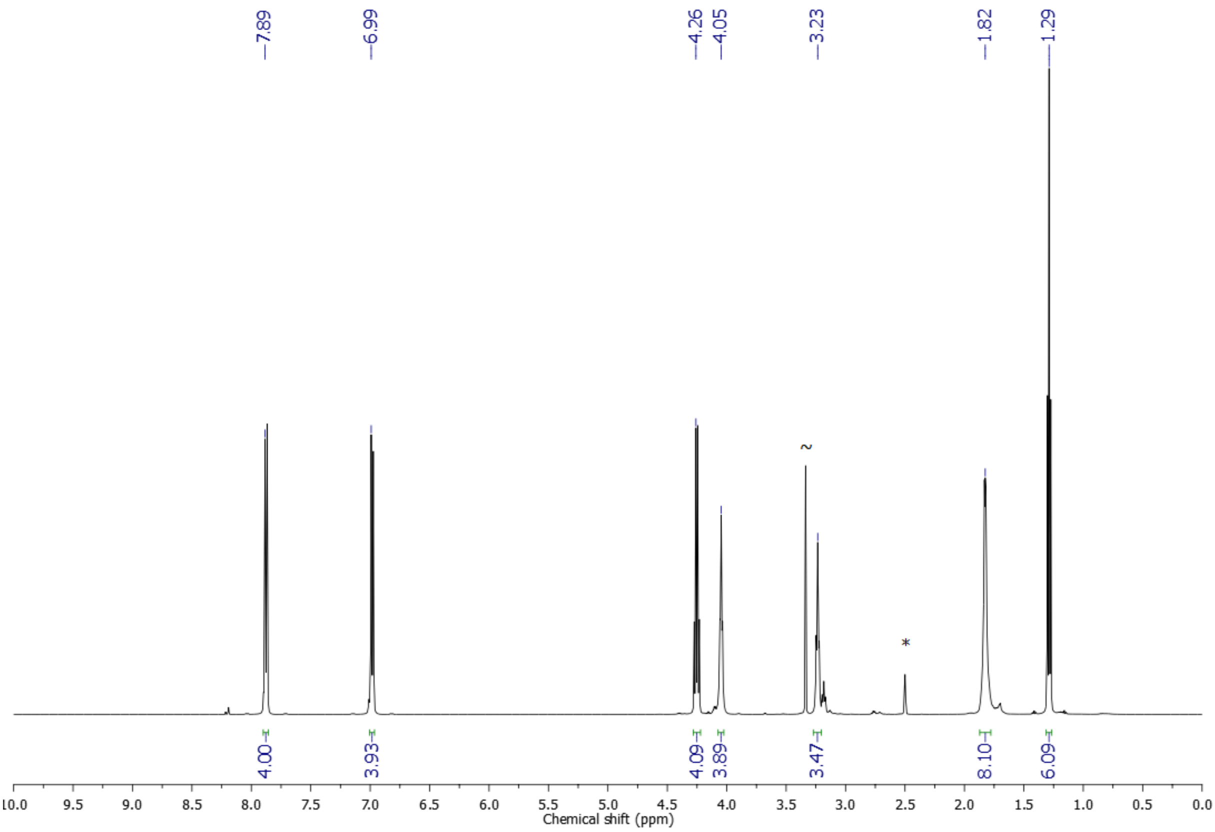

Fig. S11. ^{1}H NMR spectrum of **4** (400 MHz, DMSO-d_6, 298 K). The symbols * and ~ indicate DMSO-d_6 and water residual peaks, respectively.

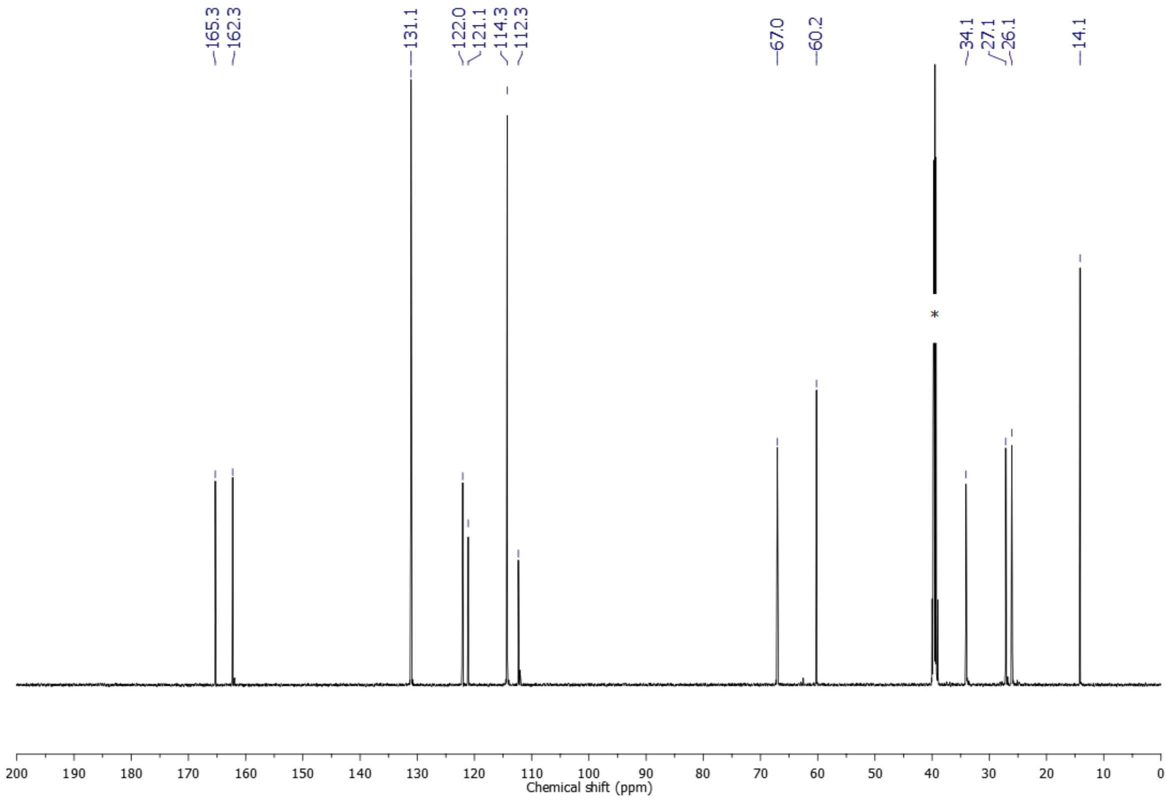

Fig. S12. ^{13}C NMR spectrum recorded for **4** (101 MHz, DMSO-d_6, 298 K). The symbol * indicates DMSO-d_6 residual peak.

1.5. NMR data of 4 macrocyclization product – an unsymmetrical porphyrazine

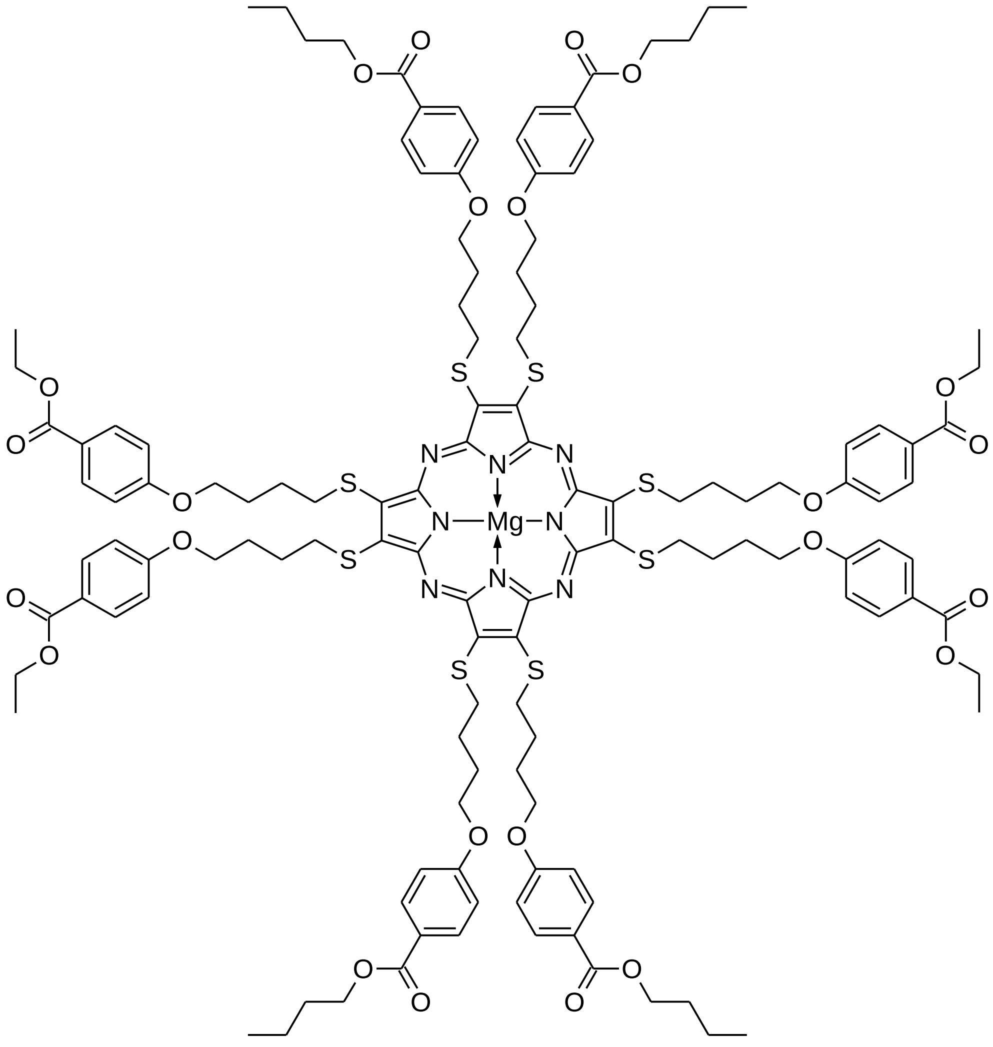

Fig. S13. Presumed structure of **4** macrocyclization product.

Table S5. ¹H and ¹³C NMR data obtained for the product of **4** macrocyclization including key correlations determined from ¹H–¹H COSY, ¹H–¹³C HSQC and ¹H–¹³C HMBC spectra.

δ_H (ppm)	Multiplicity ($J_{H–H}$ in Hz)	¹H–¹H COSY δ_H (ppm)	¹H–¹³C HSQC δ_C (ppm)	¹H–¹³C HMBC δ_C (ppm)			
8.14	d (8.5)	6.98	132.30	166.69<br>115.04	163.61	132.30	123.97
8.10	d (8.5)	6.95	132.29	166.62<br>114.99	163.59	132.29	123.97
6.98	d (8.5)	8.14	115.04	166.69<br>115.04	163.61	132.30	123.97
6.95	d (8.5)	8.10	114.99	166.62<br>114.99	163.59	132.29	123.97
4.51	s	2.26	35.67	141.61<br>27.98	68.48	68.47	29.22
4.33	q (7.0)	1.64 1.26	65.05 61.20	166.69<br>14.91	166.62<br>14.32	31.53	19.95
4.06	m	2.26	68.48 68.47	163.61<br>27.98	163.59	35.67	29.22
2.26	bs	4.06 2.26 4.51	29.22 27.98	68.48<br>27.98	68.47	35.67	29.22
1.64	m	4.33 1.36	31.53	65.05	19.95	14.32	
1.36	m	1.64 0.86	19.95	65.05	31.53	14.32	
1.26	t (7.0)	4.33	14.91	61.20			
0.86	t (7.5)	0.86	14.32	31.53	19.95		
Other carbon atoms (ppm): 158.35.							

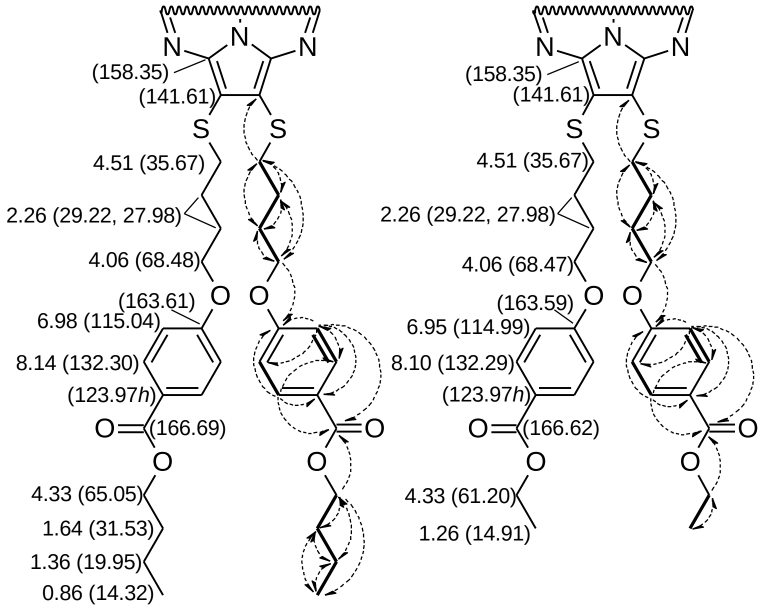

Fig. S14. ¹H and (¹³C) chemical shift values [ppm] and key correlations observed in NMR spectra of the product of **4** macrocyclization. Bold lines: ¹H–¹H COSY; Arrows: ¹H–¹³C HMBC.

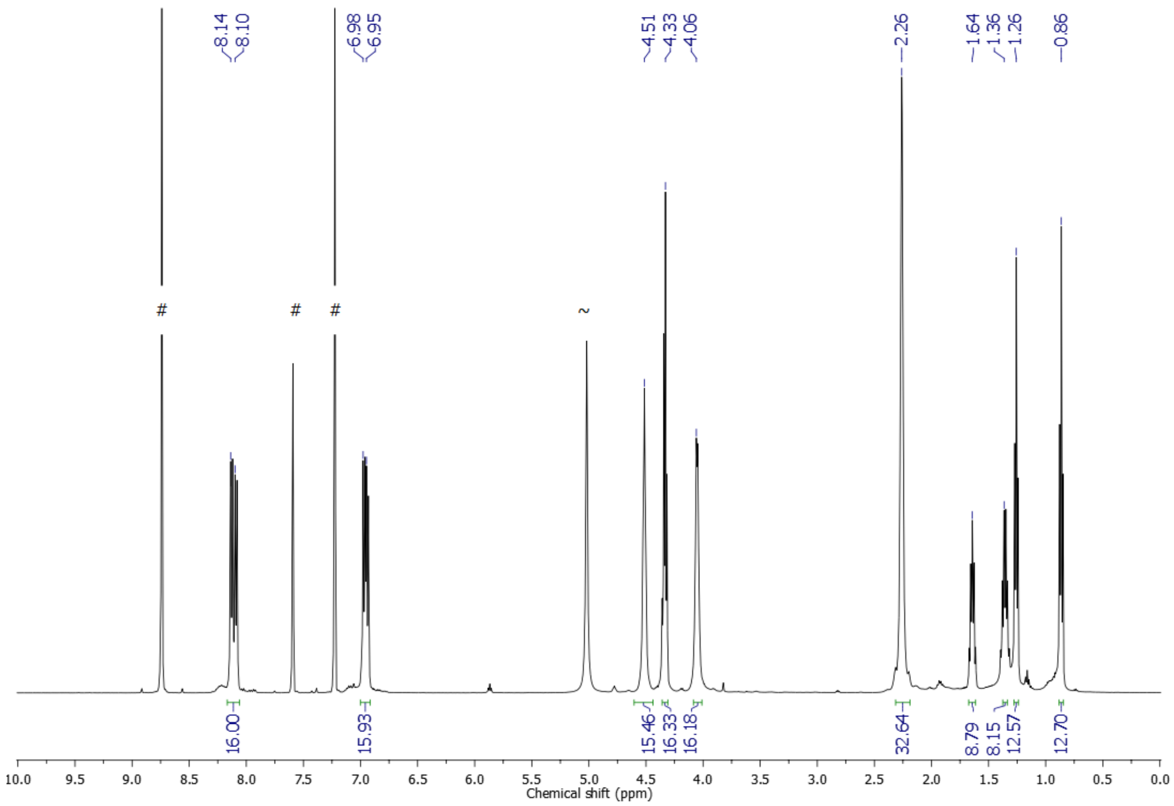

Fig. S15. ^{1}H NMR spectrum of the product of **4** macrocyclization (500 MHz, pyridine-d_5, 298 K). The symbols # and ~ indicate pyridine-d_5 and water residual peaks, respectively.

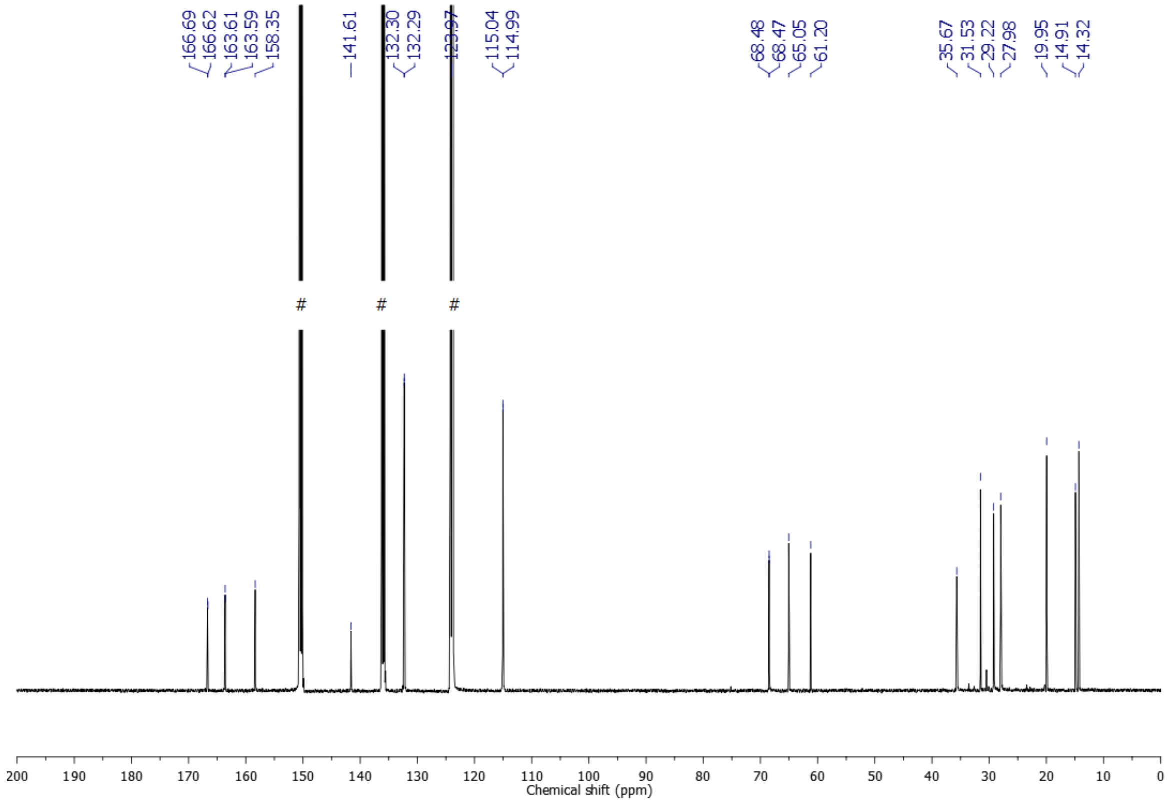

Fig. S16. ^{13}C NMR spectrum recorded for the product of **4** macrocyclization (126 MHz, pyridine-d_5, 298 K). The symbol # indicates pyridine-d_5 residual peaks.

1.6. NMR data of 5

Table S6. ^{1}H and ^{13}C NMR data obtained for **5** including key correlations determined from ^{1}H–^{1}H COSY, ^{1}H–^{13}C HSQC and ^{1}H–^{13}C HMBC spectra.

δ_H (ppm)	Multiplicity ($J_{H–H}$ in Hz)	^{1}H–^{1}H COSY δ_H (ppm)	^{1}H–^{13}C HSQC δ_C (ppm)	^{1}H–^{13}C HMBC δ_C (ppm)			
8.18	m	7.16	132.4	166.8<br>115.4	163.6	132.4	123.8
7.16	m	8.18	115.4	166.8<br>115.4	163.6	132.4	123.8
5.51	t (7.5)	2.41	61.3	29.4	26.5		
4.33	t (6.5)	1.64	65.1	166.6	31.5	20.0	
4.17	t (6.5)	2.04	68.4	163.6	29.4	26.5	
2.41	m	5.51 2.04	29.4	68.4	61.3	26.5	
2.04	m	4.17 2.41	26.5	68.4	61.4	29.4	
1.64	m	4.33 1.35	31.5	65.1	20.0	14.3	
1.35	m	1.64 0.86	20.0	65.1	31.5	14.3	
0.86	t (7.5)	1.35	14.3	31.5	20.0		

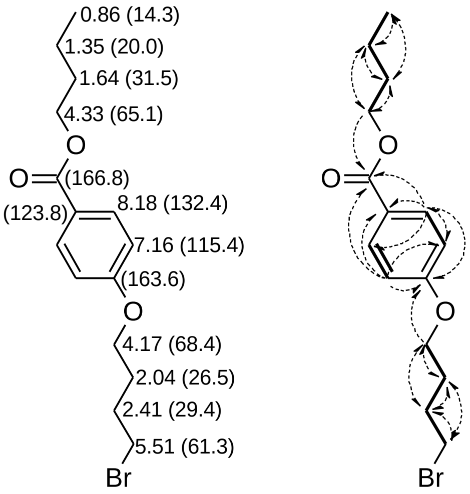

Fig. S17. ^{1}H and (^{13}C) chemical shift values [ppm] and key correlations observed in NMR spectra of **5**. Bold lines: ^{1}H–^{1}H COSY; Arrows: ^{1}H–^{13}C HMBC.

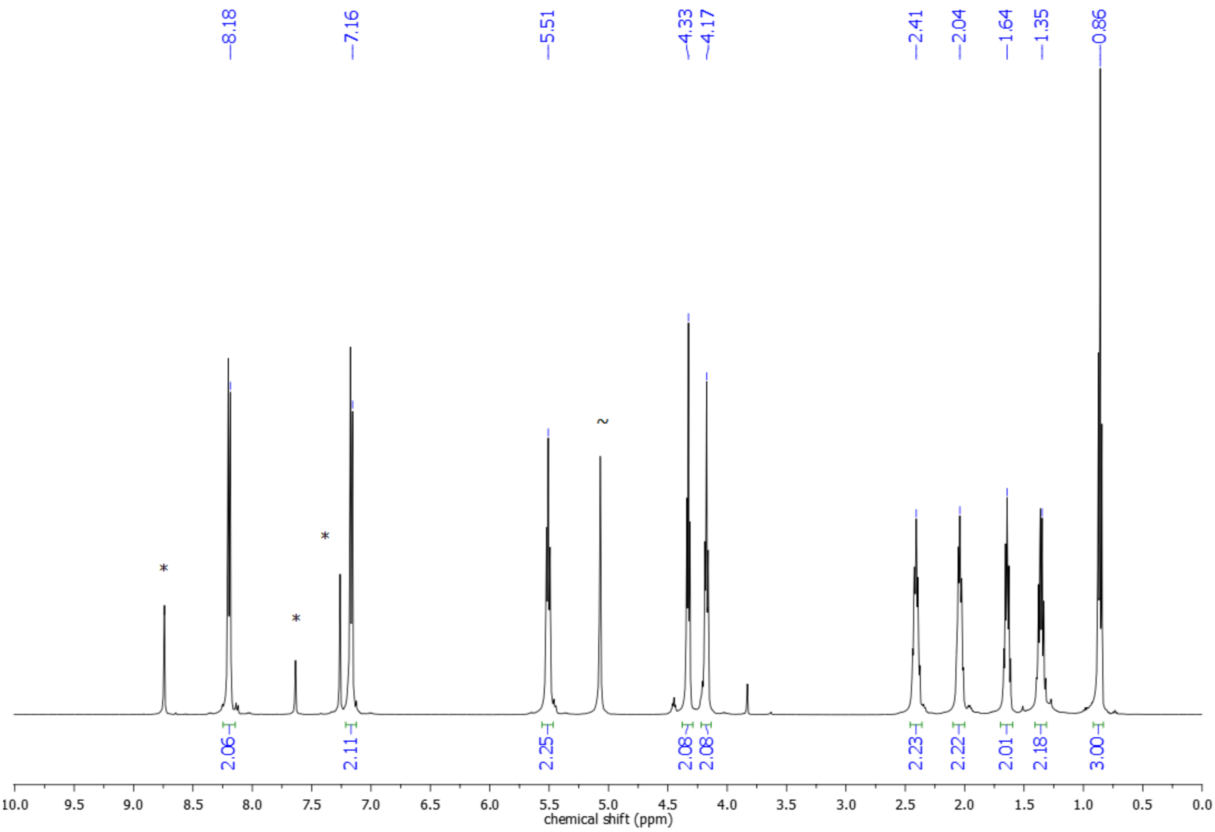

Fig. S18. ^{1}H NMR spectrum of **5** (500 MHz, pyridine-d_5, 298 K). The symbols * and ~ indicate pyridine-d_5 and water residual peaks, respectively.

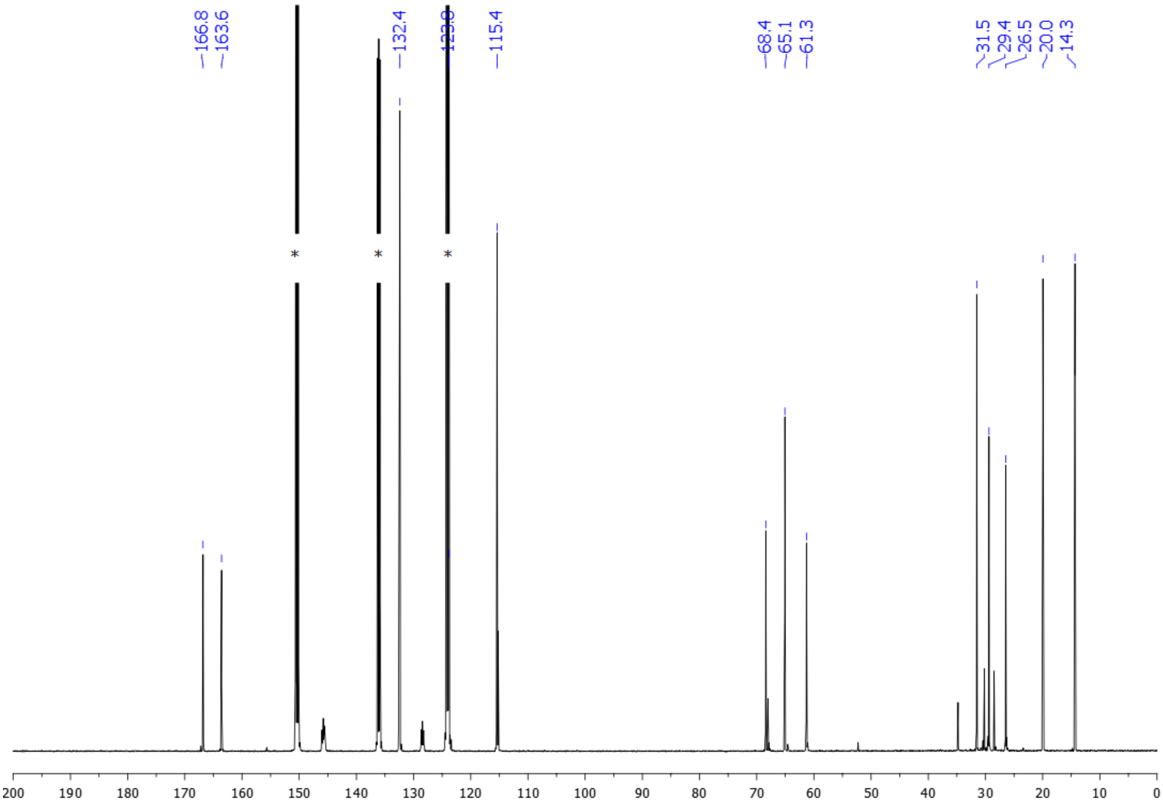

Fig. S19. ^{13}C NMR spectrum recorded for **5** (126 MHz, pyridine-d_5, 298 K). The symbol * indicates pyridine-d_5 residual peak.

1.7. NMR data of 5D

Table S7. ^{1}H and ^{13}C NMR data obtained for **5D** including key correlations determined from ^{1}H–^{1}H COSY, ^{1}H–^{13}C HSQC and ^{1}H–^{13}C HMBC spectra.

**δ_H (ppm)**	**Multiplicity ($J_{H–H}$ in Hz)**	**^{1}H–^{1}H COSY δ_H (ppm)**	**^{1}H–^{13}C HSQC δ_C (ppm)**	**^{1}H–^{13}C HMBC δ_C (ppm)**			
7.85	m	7.00	131.2	165.5	162.5	131.2	114.5
7.00	m	7.85	114.5	165.5	162.5	122.0	114.5
4.20	t (6.5)	1.64	64.0	165.5	122.0	30.3	18.8
4.09	bs	1.87	67.5	162.5	67.5	25.2	
1.87	bs	4.09	25.2	67.5	25.2		
1.64	m	4.20 1.37	30.3	64.0	18.8	13.6	
1.37	m	1.64 0.90	18.8	64.0	30.3	13.6	
0.90	t (7.5)	1.37	13.6	30.3	18.8		

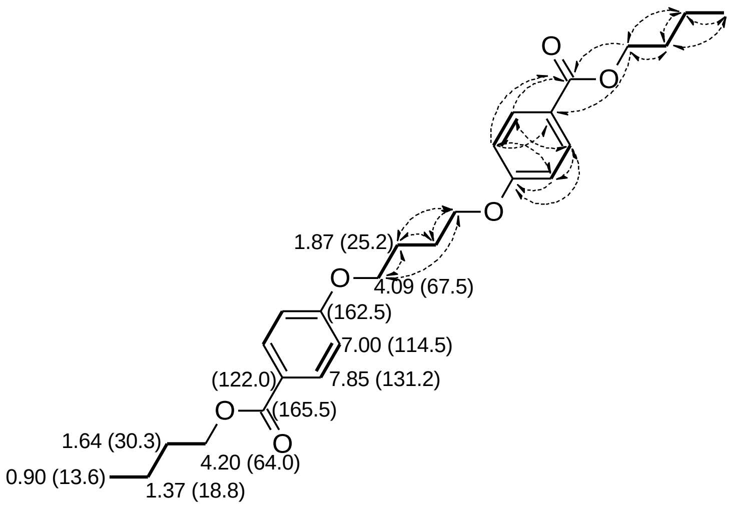

Fig. S20. ^{1}H and (^{13}C) chemical shift values [ppm] and key correlations observed in NMR spectra of **5D**. Bold lines: ^{1}H–^{1}H COSY; Arrows: ^{1}H–^{13}C HMBC.

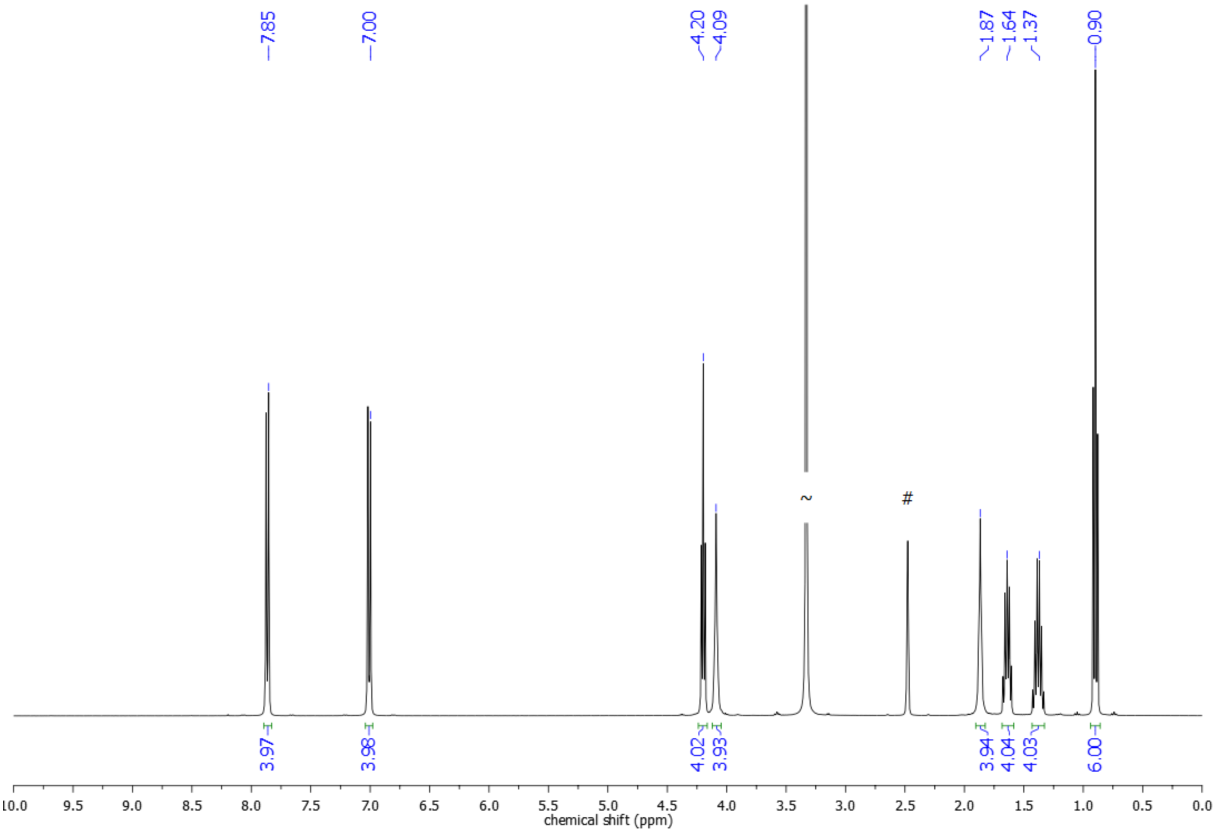

Fig. S21. ^{1}H NMR spectrum of **5D** (400 MHz, DMSO-d_6, 298 K). The symbols # and ~ indicate DMSO-d_6 and water residual peaks, respectively.

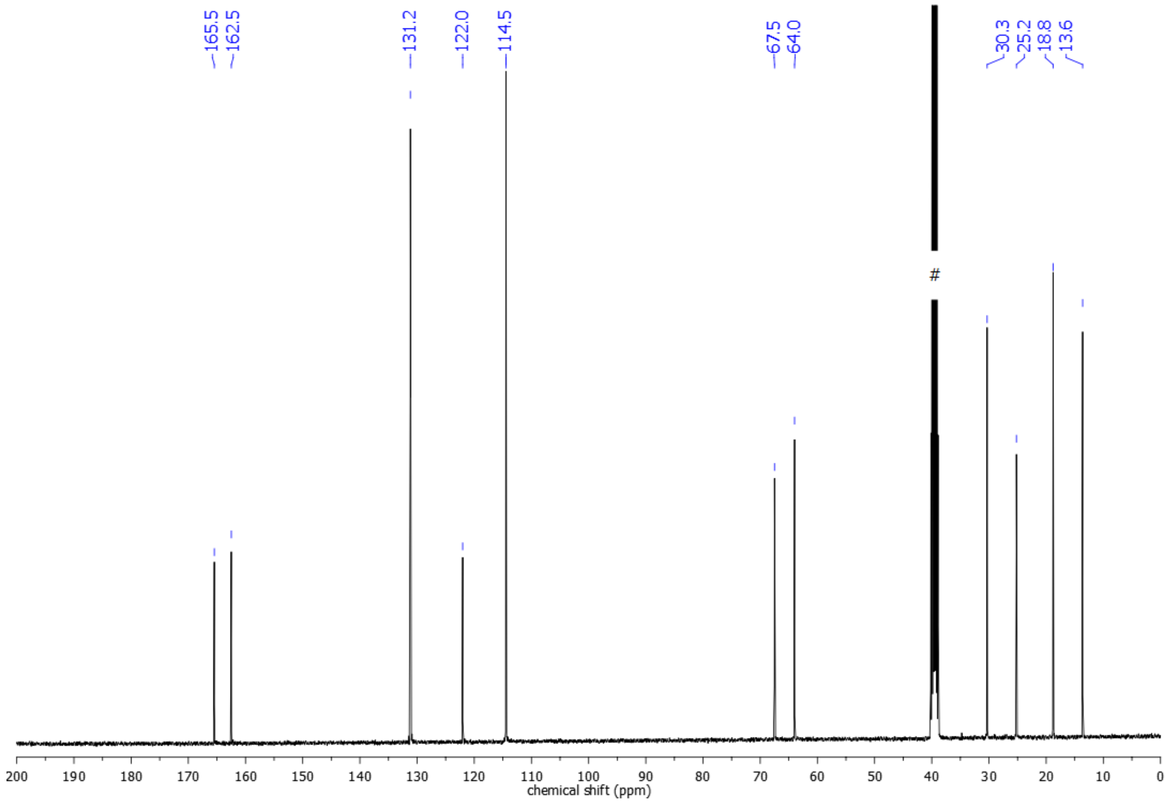

Fig. S22. ^{13}C NMR spectrum recorded for **5D** (101 MHz, DMSO-d_6, 298 K). The symbol # indicates DMSO-d_6 residual peak.

1.8. NMR data of 6

Table S8. ^{1}H and ^{13}C NMR data obtained for **6** including key correlations determined from ^{1}H–^{1}H COSY, ^{1}H–^{13}C HSQC and ^{1}H–^{13}C HMBC spectra.

δ_H (ppm)	Multiplicity ($J_{H\text{–}H}$ in Hz)	^{1}H–^{1}H COSY δ_H (ppm)	^{1}H–^{13}C HSQC δ_C (ppm)	^{1}H–^{13}C HMBC δ_C (ppm)			
7.88	m	7.00	131.0	165.4 114.3	162.3	131.0	122.0
7.00	m	7.88	114.3	165.4 114.3	162.3	131.0	122.0
4.22	t (6.5)	1.67	63.9	165.4	122.0	30.3	18.7
4.07	t (5.5)	1.83	67.1	162.3	27.1	26.1	
3.25	t (6.5)	1.83	34.1	121.2	67.1	27.1	26.1
1.83	m	4.07 3.25 1.83	27.1 26.1	67.1	34.1	27.1	26.1
1.67	m	4.22 1.40	30.3	63.9	18.7	13.6	
1.40	m	1.67 0.92	18.7	63.9	30.3	13.6	
0.92	t (7.5)	1.40	13.6	30.3	18.7		
Other carbon atoms (ppm): 112.4.							

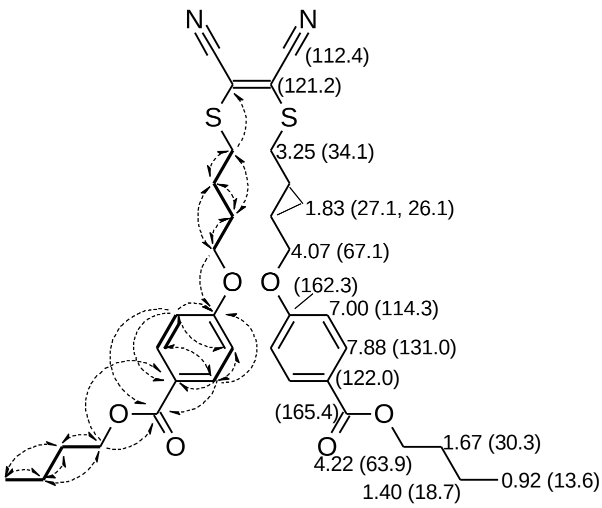

Fig. S23. ^{1}H and (^{13}C) chemical shift values [ppm] and key correlations observed in NMR spectra of **6**. Bold lines: ^{1}H–^{1}H COSY; Arrows: ^{1}H–^{13}C HMBC.

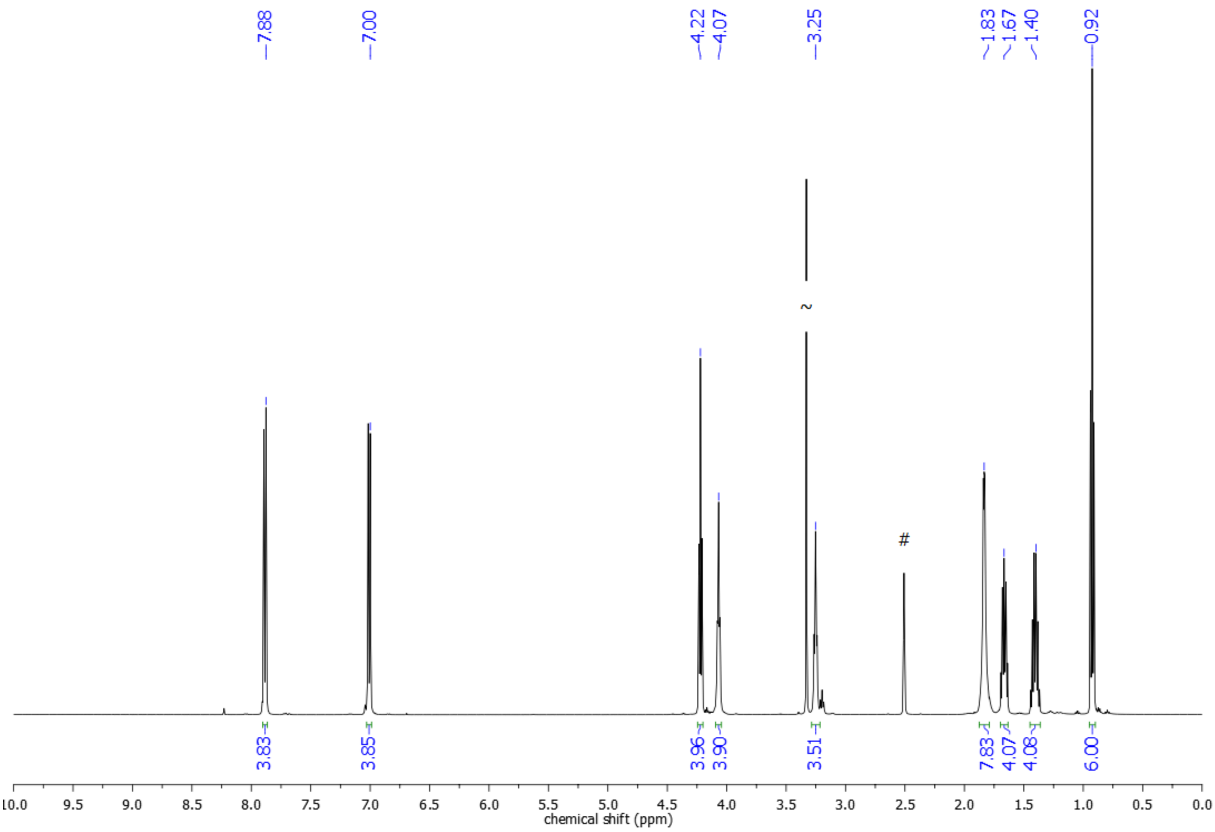

Fig. S24. ^{1}H NMR spectrum of **6** (500 MHz, DMSO-d_6, 298 K). The symbols # and ~ indicate DMSO-d_6 and water residual peaks, respectively.

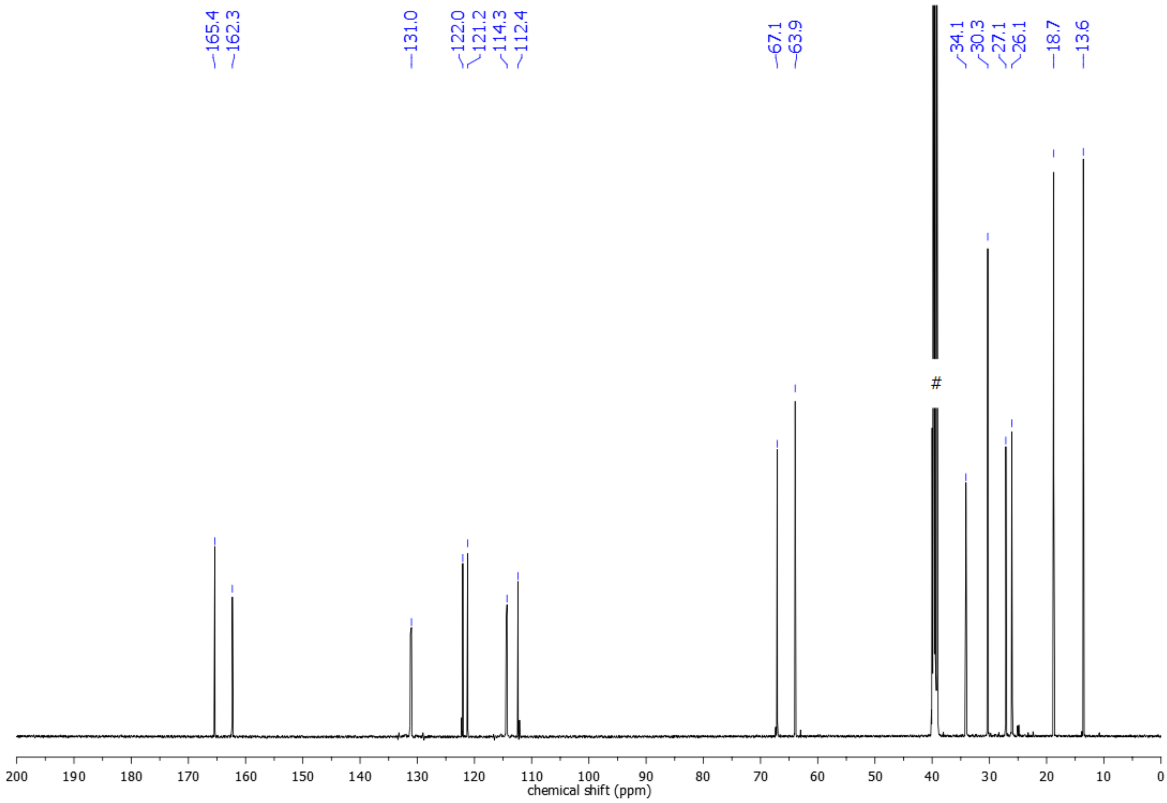

Fig. S25. ^{13}C NMR spectrum recorded for **6** (126 MHz, DMSO-d_6, 298 K). The symbol # indicates DMSO-d_6 residual peak.

1.9. NMR data of 7

Table S9. ^{1}H and ^{13}C NMR data obtained for **7** including key correlations determined from ^{1}H–^{1}H COSY, ^{1}H–^{13}C HSQC and ^{1}H–^{13}C HMBC spectra.

δ_H (ppm)	Multiplicity ($J_{H–H}$ in Hz)	^{1}H–^{1}H COSY δ_H (ppm)	^{1}H–^{13}C HSQC δ_C (ppm)	^{1}H–^{13}C HMBC δ_C (ppm)			
7.87	m	6.99	131.0	165.4 114.3	162.3	131.0	122.0
6.99	m	7.87	114.4	165.4 114.3	162.3	131.0	122.0
4.21	t (6.5)	1.66	63.9	165.4	122.0	30.3	18.7
4.06	t (5.5)	1.83	67.1	162.3	27.1	26.1	
3.24	t (6.5)	1.83	34.1	121.2	27.1	26.1	
1.83	m	4.06 3.24	27.1 26.1	67.1	34.1	27.1	26.1
1.66	m	4.21 1.39	30.3	63.9	18.7	13.6	
1.39	m	1.66 0.92	18.7	63.9	30.3	13.6	
0.92	t (7.5)	1.39	13.6	30.3	18.7		
Other carbon atoms (ppm): 112.4.							

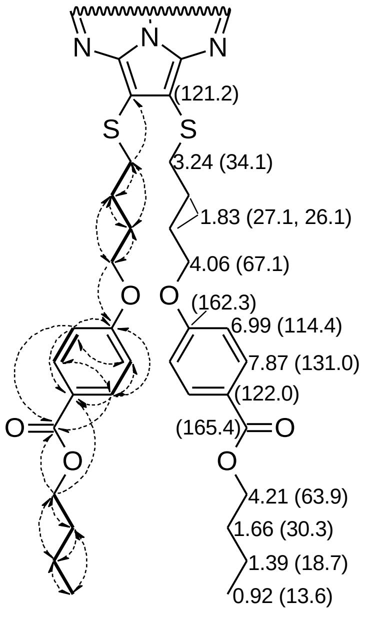

Fig. S26. ^{1}H and (^{13}C) chemical shift values [ppm] and key correlations observed in NMR spectra of **7**. Bold lines: ^{1}H–^{1}H COSY; Arrows: ^{1}H–^{13}C HMBC.

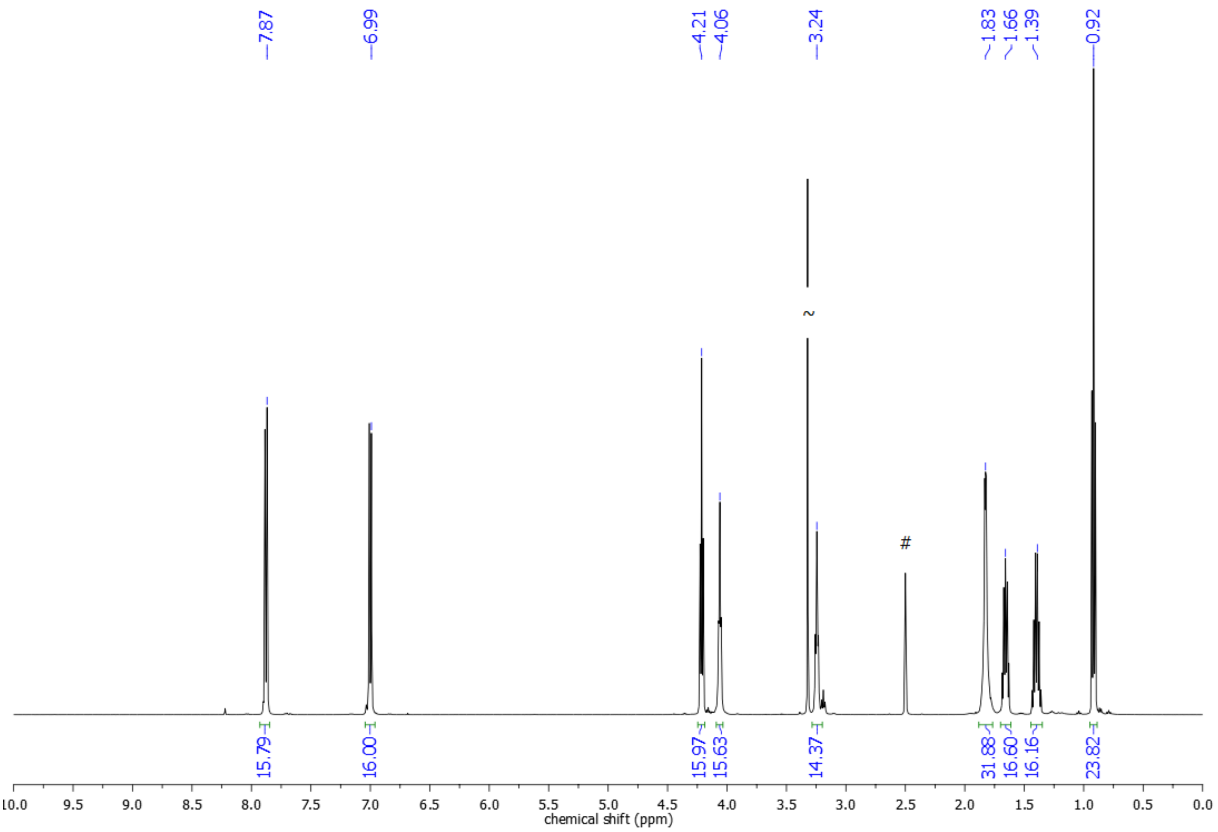

Fig. S27. ^{1}H NMR spectrum of **7** (500 MHz, DMSO-d_6, 298 K). The symbols # and ~ indicate DMSO-d_6 and water residual peaks, respectively.

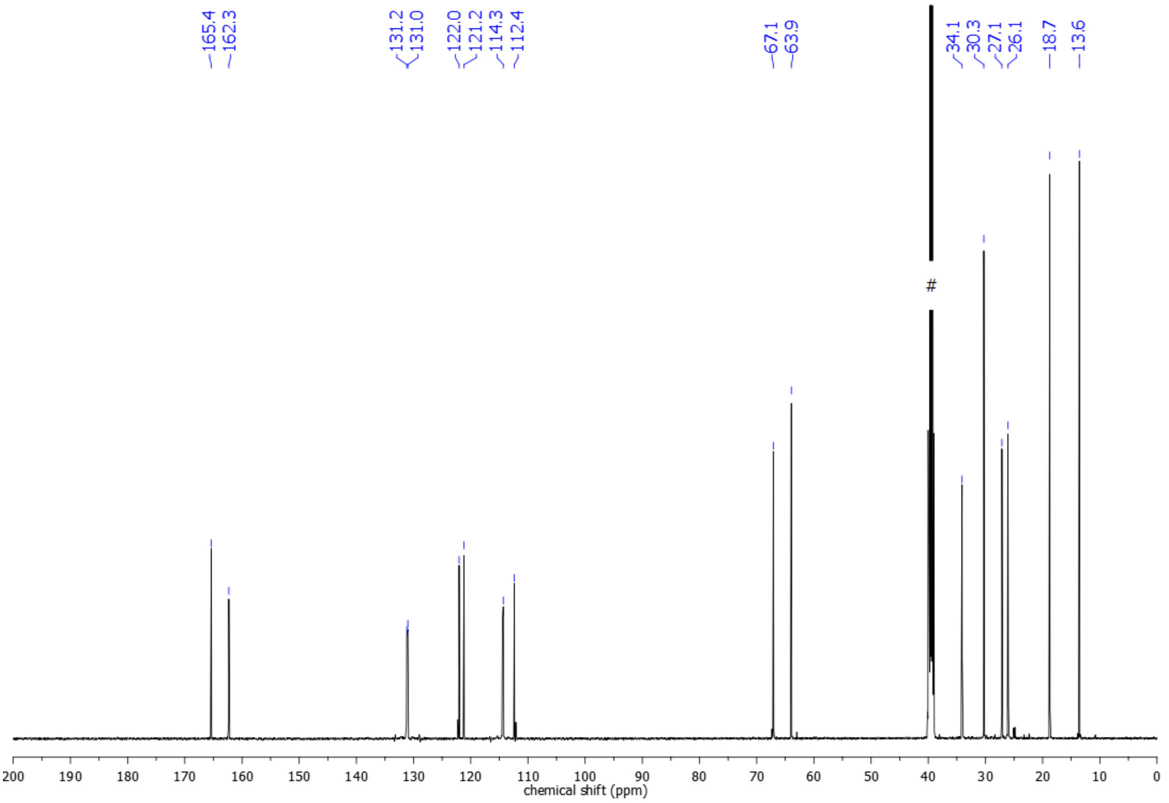

Fig. S28. ^{13}C NMR spectrum recorded for **7** (126 MHz, DMSO-d_6, 298 K). The symbol # indicates DMSO-d_6 residual peak.

1.10. NMR data of 8

Table S10. ^{1}H and ^{13}C NMR data obtained for **8** including key correlations determined from ^{1}H–^{1}H COSY, ^{1}H–^{13}C HSQC and ^{1}H–^{13}C HMBC spectra.

δ_H (ppm)	Multiplicity (J_{H-H} in Hz)	^{1}H–^{1}H COSY δ_H (ppm)	^{1}H–^{13}C HSQC δ_C (ppm)	^{1}H–^{13}C HMBC δ_C (ppm)			
7.85	m	7.00	131.2	165.4	162.4	131.2	114.4
7.59	s	7.59	120.5	154.8	120.5	113.6	
7.00	m	7.85	114.4	165.4	162.4	122.0	114.4
4.20	m	1.89 1.65	69.4 64.0	165.4 18.8	154.8	30.5	25.0
4.12	bs	1.89	67.4	162.4	25.0		
1.89	s	4.20 4.12	25.0	69.4	67.4	25.0	
1.65	m	4.20 1.38	30.3	64.0	18.8	13.6	
1.38	m	1.65 0.90	18.8	64.0	30.3	13.6	
0.90	t (7.5)	1.38	13.6	30.3	18.8		
Other carbon atoms (ppm): 102.8.							

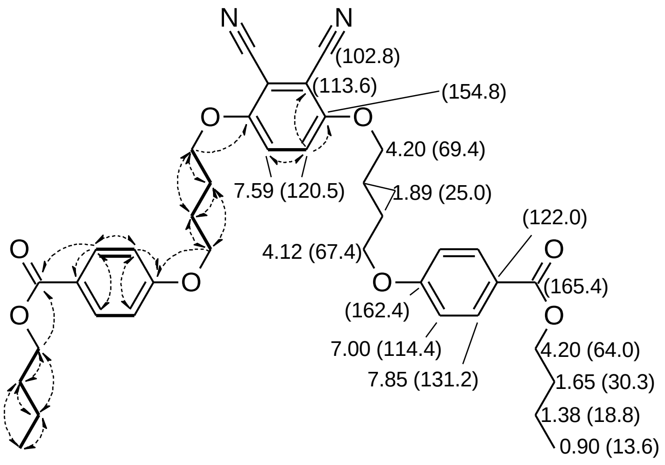

Fig. S29. ^{1}H and (^{13}C) chemical shift values [ppm] and key correlations observed in NMR spectra of **8**. Bold lines: ^{1}H–^{1}H COSY; Arrows: ^{1}H–^{13}C HMBC.

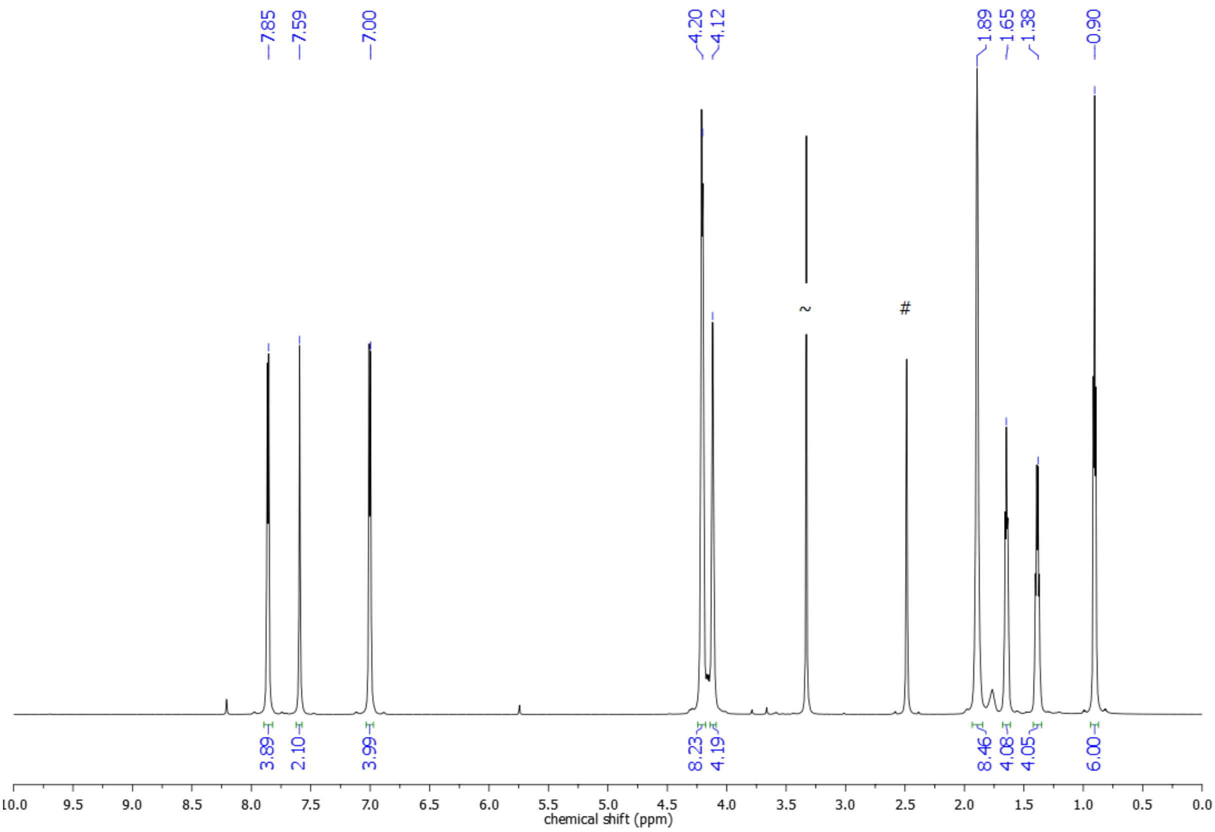

Fig. S30. ^{1}H NMR spectrum of **8** (700 MHz, DMSO-d_6, 298 K). The symbols # and ~ indicate DMSO-d_6 and water residual peaks, respectively.

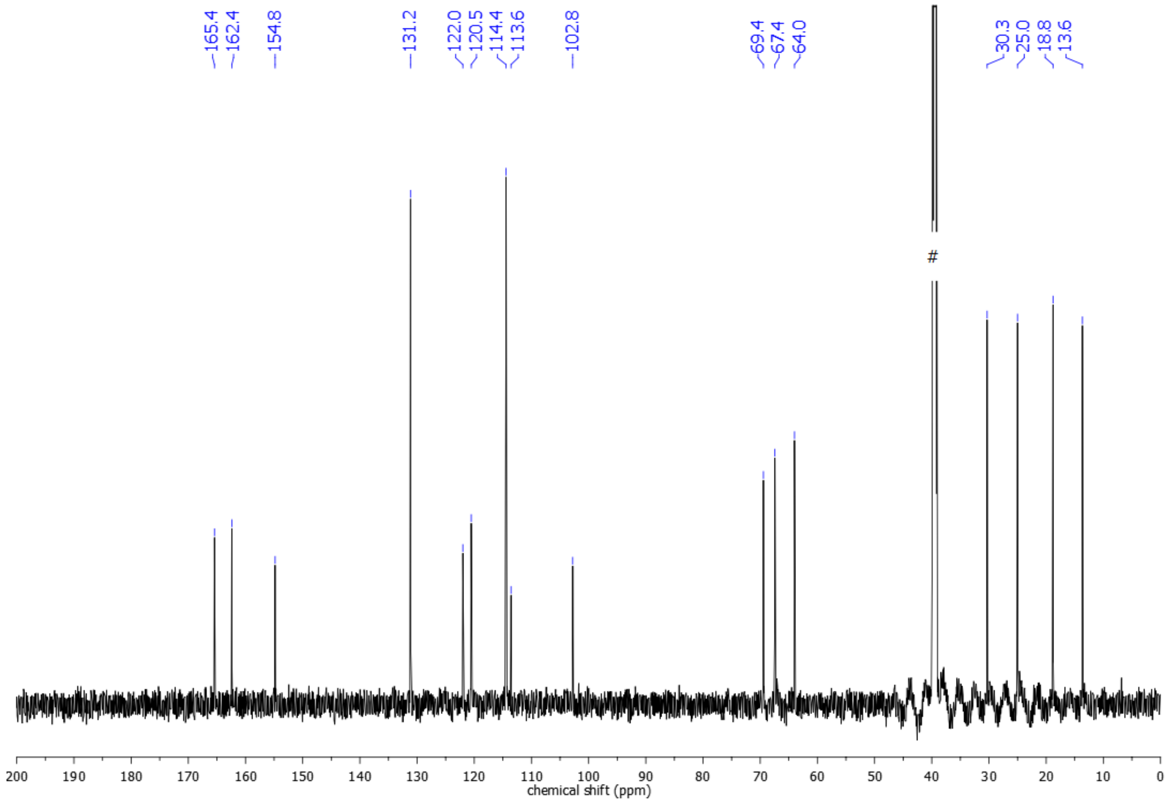

Fig. S31. ^{13}C NMR spectrum recorded for **8** (176 MHz, DMSO-d_6, 298 K). The symbol # indicates DMSO-d_6 residual peak.

1.11. HPLC data of 3

Analytical HPLC was carried out on an Agilent 1200 instrument equipped with a DAD detector. The chromatographic separation was achieved on octadecylsilane coated column, 150 mm × 4.6 mm, 5 µm (Eclipse XDB-C18, Agilent) using a linear gradient conditions at a flow rate of 1.0 mL/min in different configurations. Separations was performed at 25°C.

1.11.1. Phases configuration 1

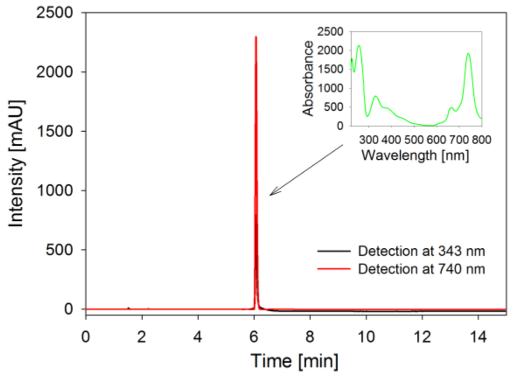

Mobile phase

Time [min]	MeOH	CH_2Cl_2
0	100	0
3	100	0
4	0	100
15	0	100

Flow	1.0 ml/min
Temperature	25°C
Column	Agilent, Eclipse XDB-C18
	150 mm · 4.6 mm, 5 µm

Detection at λ = 343 nm

Signal	Retention time [min]	Area	Content [%]
1	1.53	31.9	0.90
2	1.60	8.5	0.24
3	2.22	10.3	0.29
4	6.07	3517.2	98.58

Detection at λ = 740 nm

Signal	Retention time [min]	Area	Content [%]
1	5.60	24.6	0.29
2	6.07	8497.3	99.71

1.11.2. Phases configuration 2

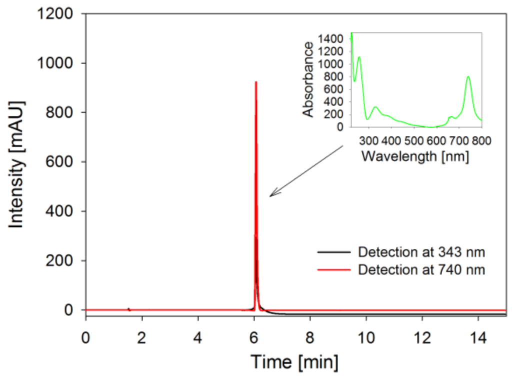

Mobile phase

Time [min]	MeOH	CH_2Cl_2	THF		
				Flow	1.0 ml/min
0	100	0	0	Temperature	25°C
3	100	0	0	Column	Agilent, Eclipse XDB-C18
4	0	95	5		150 mm · 4.6 mm, 5 µm
15	0	95	5		

Detection at λ = 343 nm

Signal	Retention time [min]	Area	Content [%]
1	1.53	20.3	1.21
2	6.07	1652.2	98.79

Detection at λ = 740 nm

Signal	Retention time [min]	Area	Content [%]
1	5.60	26.9	0.79
2	6.07	3393.1	99.21

1.11.3. Phases configuration 3

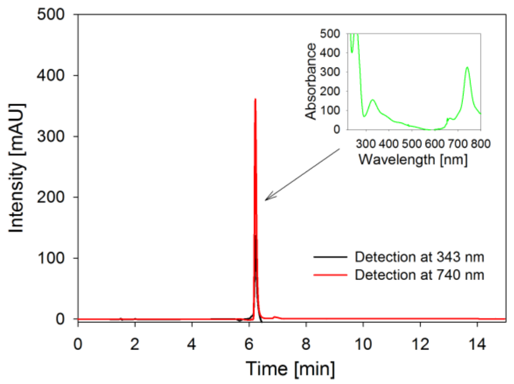

Mobile phase

Time [min]	MeOH	CH_2Cl_2
0	90	10
3	90	10
4	10	90
15	10	90

Flow	1.0 ml/min
Temperature	25°C
Column	Agilent, Eclipse XDB-C18
	150 mm · 4.6 mm, 5 µm

Detection at λ = 343 nm

Signal	Retention time [min]	Area	Content [%]
1	1.50	6.9	0.62
2	2.01	5.6	0.51
3	6.22	1080.1	97.24
4	6.89	18.1	1.63

Detection at λ = 740 nm

Signal	Retention time [min]	Area	Content [%]
1	5.65	17.5	1.00
2	6.22	1706.5	97.35
3	6.89	29.0	1.65

1.12. HPLC data of 7

Analytical HPLC was carried out on an Agilent 1200 instrument equipped with a DAD detector. The chromatographic separation was achieved on octadecylsilane coated column, 150 mm × 4.6 mm, 5 µm (Eclipse XDB-C18, Agilent) using a linear gradient conditions at a flow rate of 1.0 mL/min in different configurations. Separations was performed at 25°C.

1.12.1. Phases configuration 1

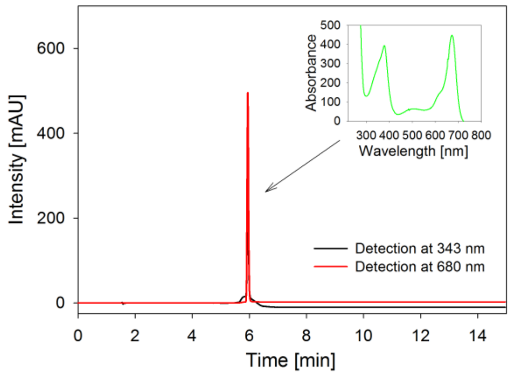

Mobile phase

Time [min]	MeOH	THF
0	100	0
3	100	0
4	0	100
15	0	100

Flow	1.0 ml/min
Temperature	25°C
Column	Agilent, Eclipse XDB-C18<br>150 mm · 4.6 mm, 5 µm

Detection at λ = 343 nm

Signal	Retention time [min]	Area	Content [%]
1	1.54	7.8	0.49
2	5.95	1574.0	99.51

Detection at λ = 680 nm

Signal	Retention time [min]	Area	Content [%]
1	5.94	1675.2	100.00

1.12.2. Phases configuration 2

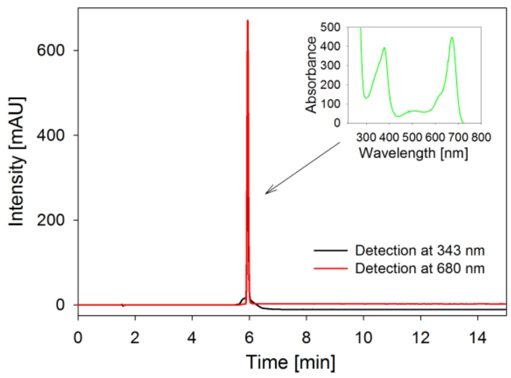

Mobile phase

Time [min]	MeOH	THF	CH_2Cl_2
0	100	0	0
3	100	0	0
4	0	95	5
15	0	95	5

Flow	1.0 ml/min
Temperature	25°C
Column	Agilent, Eclipse XDB-C18
	150 mm · 4.6 mm, 5 µm

Detection at λ = 343 nm

Signal	Retention time [min]	Area	Content [%]
1	1.53	7.3	0.37
2	5.94	1943.3	99.63

Detection at λ = 680 nm

Signal	Retention time [min]	Area	Content [%]
1	5.94	2261.9	100.00

1.12.3. Phases configuration 3

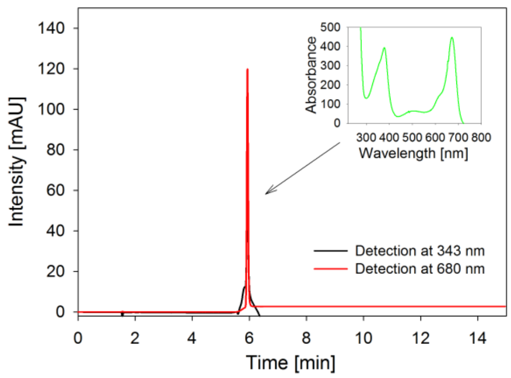

Mobile phase

Time [min]	MeOH	THF
0	95	5
3	95	5
4	5	95
15	5	95

Flow	1.0 ml/min
Temperature	25°C
Column	Agilent, Eclipse XDB-C18
	150 mm · 4.6 mm, 5 µm

Detection at λ = 343 nm

Signal	Retention time [min]	Area	Content [%]
1	1.59	7.5	1.05
2	5.93	704.3	98.95

Detection at λ = 680 nm

Signal	Retention time [min]	Area	Content [%]
1	5.93	417.8	100.00

2. Synthetic attempts towards A_3B type macrocycles.

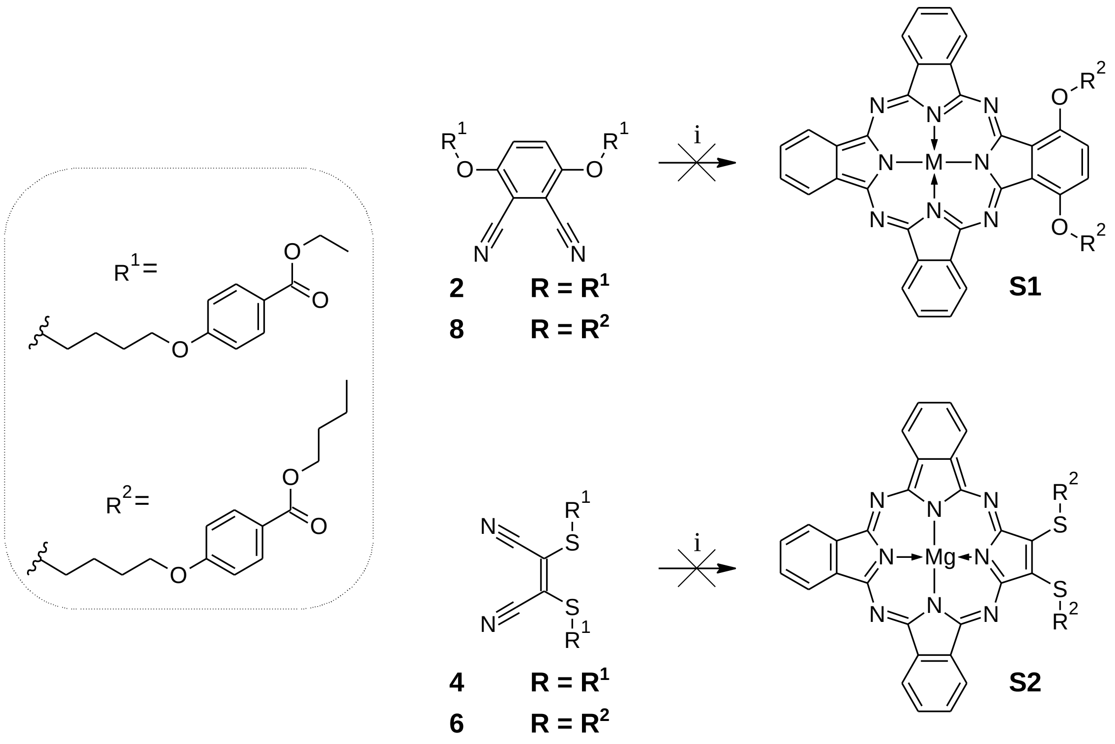

Fig. S32. Synthetic approaches towards A_3B macrocycles: (i) 1,2-dicyanobenzene (tenfold excess), $(BuO)_2Mg$, BuOH, reflux, 20h.

S1 synthesis:

Magnesium turnings (222 mg, 9.16 mmol) were stirred in refluxing butan-1-ol with catalytic amount of iodine for 3 h. After cooling, **4** (1000 mg, 1.665 mmol) and 1,2-dicyanobenzene (2133 mg, 16.65 mmol) were added and the reaction mixture was stirred under reflux for another 20 h in inert gas atmosphere.

After cooling, the reaction mixture was poured on water:methanol 1:1 (v/v) mixture. Formed precipitate was filtered and washed with methanol. After drying, green product was dissolved in DCM and purified by column chromatography using: silica gel and eluents DCM, DCM/MeOH 100:1 (v/v), DCM/MeOH 50:1 (v/v), hexane/ethyl acetate 7:2 (v/v); C_{18}-reversed phase silica gel and eluents H_2O/MeOH 3:1 (v/v), MeOH, DCM/MeOH 20:1 (v/v), DCM/MeOH 1:3 (v/v) to give a deep green film. The methods presented below indicated the presence of macrocyclic isomers.

HRMS (MALDI) *m/z* found: 1064.4164, $[M]^+$ $C_{62}H_{56}MgN_8O_8$ requires 1064,4072; 1065.4261, $[M+H]^+$ $C_{62}H_{57}MgN_8O_8$ requires 1065,4150; 1069.3370, $[M+Na]^+$ $C_{58}H_{54}MgN_8O_6S_2Na$ requires 1069,3356. Also A_2B_2 and AB_3 isomers were formed, detectable by MS (found: 1592.6930, $[ABAB]^+$ or $[A_2B_2]^+$ $C_{92}H_{96}MgN_8O_{16}$ require 1592.6795; 1593.6926, $[ABAB+H]^+$ or $[A_2B_2+H]^+$ $C_{92}H_{96}MgN_8O_{16}$ require 1593.6873).

S2 synthesis:

Magnesium turnings (120 mg, 4.946 mmol) were stirred in refluxing butan-1-ol with catalytic amount of iodine for 3 h. After cooling, **2** (524 mg, 0.899 mmol) and 1,2-dicyanobenzene (1152 mg, 8.992

mmol) were added and the reaction mixture was stirred under reflux for another 20 h in inert gas atmosphere.

After cooling, solvent was evaporated, and the obtained crude residue was purified by column chromatography using: silica gel and eluents DCM, DCM/MeOH 100:1 (v/v), DCM/MeOH 50:1 (v/v), hexane/ethyl acetate 7:2 (v/v); C_{18}-reversed phase silica gel and eluents H_2O/MeOH 3:1 (v/v), MeOH, DCM/MeOH 20:1 (v/v), DCM/MeOH 1:3 (v/v) to give a a deep blue film. The methods presented below indicated the presence of macrocyclic isomers.

HRMS (MALDI) *m/z* found: 1046.3588, $[M]^+$ $C_{58}H_{54}MgN_8O_6S_2$ requires 1046,3458; 1047.3581, $[M+H]^+$ $C_{58}H_{55}MgN_8O_6S_2$ requires 1047.3536; 1069.3370, $[M+Na]^+$ $C_{58}H_{54}MgN_8O_6S_2Na$ requires 1069.3356. Also A_2B_2 and AB_3 isomers were formed, as detected by MS (found: 1557.5634, $[ABAB+H]^+$ or $[A_2B_2+H]^+$ $C_{84}H_{92}MgN_8O_{12}S_4$ require 1557.5646; 2069.7796, $[AB_3+H]^+$ $C_{110}H_{131}MgN_8O_{18}S_6$ requires 2067.7756).
